# To be well or not to be well: compositional associations of physical activity, sedentary behaviour and sleep with mental well-being in Flemish adults aged 55+ years

**DOI:** 10.1186/s44167-023-00019-3

**Published:** 2023-05-01

**Authors:** Julie Vanderlinden, Gregory J. H. Biddle, Filip Boen, Jannique G. Z. van Uffelen

**Affiliations:** 1 Physical Activity, Sports & Health Research Group, Department of Movement Sciences, KU Leuven, 3000 Leuven, Belgium; 2 Department of Health Care, Odisee University of Applied Sciences, 1000 Brussels, Belgium; 3 School of Sport, Exercise and Health Sciences, Loughborough University, Loughborough, LE11 3TU UK; 4 Diabetes Research Centre, University of Leicester, Leicester, LE5 4PW UK; 5 NIHR Leicester Biomedical Research Centre, Leicester, LE5 4PW UK

**Keywords:** Well-being, Mental health, Physical activity, Sedentary behaviour, Sleep, Public health, Health promotion, Lifestyle, Ageing

## Abstract

**Background:**

Well-being is a key aspect for healthy ageing and there is an established association between physical activity and well-being in ageing adults. Despite the recent interest in physical activity as part of a 24-h continuum also including sedentary behaviour and sleep, there is a lack of studies examining the link between these 24-h behaviours and well-being in older adults. Therefore, the aim of this study was twofold: (1) to describe 24-h behaviours and their associations with mental well-being in community dwelling adults aged 55+ years; and (2) to examine the theoretical changes in mental well-being if time were reallocated from one behaviour to another.

**Methods:**

This was a cross-sectional study (n = 410). Daily time spent in sedentary behaviour, light and moderate-vigorous physical activity, and sleep was assessed using wrist-worn accelerometers during 6 days. Mental well-being was assessed using the 14-item Warwick-Edinburgh Mental Well-being Scale (WEMWBS). The association of 24-h behaviours and well-being was examined using crude and adjusted linear regression models with compositional data analysis procedures (aim 1). Associations between reallocations of five-minute intervals from five to 60 min between these behaviours and well-being were modelled using compositional isotemporal substitutions (aim 2).

**Results:**

Mean age (SD) was 71.3 (6.3) years and 71% were female. In 24-h, participants spent 5.66 h asleep, 13.88 h sedentary, 2.58 h in light intensity and 1,89 h in moderate-vigorous intensity physical activity. There were no statistically significant associations between 24-h behaviours and mental well-being in fully adjusted models (aim 1). Reallocations up to 30 min were not significantly associated with changes in well-being (aim 2). There were some statistically significant theoretical changes of up to 2 points on the WEMWBS with substitutions larger than 30 min.

**Discussion:**

24-h behaviours and time reallocations between behaviours were not associated with better or worse well-being in community dwelling adults aged 55+ years. Considering well-being as a key aspect for healthy ageing, future public health research should continue to examine physical activity (both light and moderate-to-vigorous), sedentary behaviour, as well as sleep as a part of the 24-h continuum and its associations with mental health outcomes in older adults.

*Trial registration* This trial was registered at ClinicalTrials.gov on 3th July, 2018 (Trial registration NCT03576209)

**Supplementary Information:**

The online version contains supplementary material available at 10.1186/s44167-023-00019-3.

## Introduction

One of the success stories of public health is that people are living longer. For example, in Europe, life expectancy has steadily increased over the past two decades from 74 to 78 years for men and from 81 to 84 years for women [1]. However, not all of these additional years are spent in good health. Ageing is typically associated with declines in physical function, aspects of cognitive function, as well as an increased prevalence of chronic conditions [2]. Consequently, healthy life expectancy in Europe is 63.5 years and 64.5 years and equates to 81% and 77% of the total life expectancy of men and women respectively. ‘Healthy life expectancy’ is typically based on morbidity and mortality data and does not take well-being into account [3]. However, well-being is a key aspect in the definition of healthy ageing by the World Health Organization (WHO), namely ‘to develop and maintain the functional ability that enables well-being in older age’ [4].

According to the WHO [5] well-being is “a positive state experienced by individuals and societies. Similar to health, well-being is a resource for daily life and is determined by social, economic and environmental conditions” (p. 10). Interestingly, the relation between age and well-being is U-shaped: the lowest levels are typically seen at mid-age, with increased well-being as people get older, even if their health declines [6, 7]. This relation clearly indicates that although health is an important aspect of well-being, well-being encompasses more than health alone. Well-being is indeed a multi-faceted concept, including at least three aspects: (a) evaluative well-being, referring to life satisfaction, (b) hedonic well-being, referring to everyday feelings or moods (positive and negative affect) and (c) eudaimonic well-being, referring to a sense of purpose and meaning in life) [6, 7]. Unlike morbidity and mortality, well-being is based on cognitive and affective self-evaluations of a person’s life. Well-being therefore is a meaningful outcome for people [7]. Therefore, examining measures of well-being is in line with the need to not only focus on function and morbidity, but to have an additional focus on other relevant outcomes that affect the ageing process [8].

Determinants of well-being include non-modifiable factors, such as biological factors [6], factors that are not straightforward to change, such as socioeconomic status [9], as well as modifiable lifestyle behaviours. Physical activity (PA) has been identified as an important lifestyle behaviour connecting well-being and health [10]. Indeed, physical activity has been consistently associated with better mental well-being in mid-aged and older adults [11–13]. For example, based on a systematic review, summarising 42 papers on physical activity and quality of life in adults aged 60+ years, it was concluded that the more physical active older adults are, the higher their well-being [13]. However, most of the studies on physical activity and well-being focus on moderate to vigorous physical activity (MVPA) as a single factor, not considering light intensity physical activity (LPA), sedentary behaviour (SB) and sleep.

Recently, there has been a shift in physical activity epidemiology from a focus on examining only moderate-to-high intensity physical activity, to physical activity as part of a 24-h paradigm [14, 15]. This paradigm also includes sedentary behaviour, different intensities of physical activity and sleep. Data from mid-aged adults participating in the British Cohort Study indicated that ST was only associated with reduced mental well-being in participants with low levels of MVPA [12]. While the evidence for an association of sedentary behaviour with well-being in older adults is indicated to be inconclusive in a systematic review of studies in adults aged 60+ years [16], sleep has been identified as a critical determinant of well-being in this population group in the American Academy of Sleep Medicine position statement [17]. Because sedentary behaviour, physical activity and sleep are part of the 24-h continuum, it is imperative to use appropriate statistical methods that consider these behaviours as part of a mutually exclusive and exhaustive 24-h day [14, 15]. In doing so, it will become possible to not only examine associations of these behaviours with well-being, but also their associations with time reallocations from one behaviour to another.

Previous studies that examined 24-h behaviours and mental health in older adults predominantly focussed on mental health outcomes such as anxiety and depression [18–20], rather than mental well-being. There are only few studies examining associations of 24-h patterns with mental well-being specifically in different population groups, including adolescents [21], adults [22] and adult office workers [23] and retirees [24]. These studies used both self-reported and device-based measures of SB, PA and sleep with conflicting results. There were positive associations with mental well-being when time was reallocated from self-reported SB to MVPA or sleep, but there were no associations when time was reallocated from self-reported MVPA to sleep in adolescents [21]. Similarly, in retirees, the change in overall activity composition was also not related to changes in wellbeing [24]. Furthermore, there were no reported associations between reallocated device-measured SB, PA and sleep and self-reported mental well-being in adults [22], or between device-measured SB, PA and self-reported sleep and mental well-being in office workers [23]. Cabanas-Sánchez et al. [20] did examine associations of device-measured 24-h behaviours with happiness in adults aged 65+ years. Not only moderate-vigorous physical activity time, but also replacing half an hour of sleep, sedentary behaviour or light intensity physical activity with moderate-vigorous intensity activity, was positively associated with happiness [20]. The identified lack of studies examining 24-h behaviours and mental well-being in older adults is a clear gap in the literature given the importance of well-being as an essential aspect of healthy ageing [4]. Furthermore, with several countries now issuing 24-h guidelines [25–27], it is the natural progression of the field to examine associations of 24-h patterns with different health outcomes, including wellbeing. Therefore, the aim of this study is twofold. The first aim is to describe the 24-h behaviours (sedentary behaviour, physical activity and sleep) and their associations with mental well-being in community dwelling adults aged 55+ years. The second aim is to explore the theoretical changes in mental well-being if time were reallocated from one behaviour (SB, PA or sleep) to another.

## Methods

### Study sample and design

Cross-sectional data were gathered from community-dwelling older adults aged 55+ years from July 2018 to July 2019. These adults were recruited by a community service organization that provides physical and socio-cultural activities for older adults in Flanders (OKRA SPORT+). The ethics committee of UZ Leuven (Belgium) granted approval for this study (Ref. no: S61581). All participants were informed about the study and granted their written informed consent.

### Measurement

Data were collected using self-administered questionnaires (demographics and Warwick-Edinburgh Mental Well-being Scale (WEMWBS) and accelerometry (physical activity, sedentary time and total sleep time).

### Demographics and health related variables

Demographic variables (age in years, gender [male, female], education [low, medium, high], marital status [married/cohabitant, Single], professional status [active/inactive]), as well as general health information, were collected by means of a questionnaire. The definition of categories for education are in line with the International Standard Classification of Education (ISCED) [28]. Regarding health-related variables, all participants were asked to indicate whether they suffered from chronic conditions, such as cancer, cardiovascular disease, diabetes or metabolic/intestinal disease, arthritis or pain, respiratory diseases, cognitive, psychiatric or other illnesses. The variable chronic conditions was then recoded into a dichotomous variable.

### Mental well-being

The Warwick-Edinburgh Mental Well-being Scale (WEMWBS) was funded by the Scottish Government National Programme for Improving Mental Health and Well-being, commissioned by NHS Health Scotland, developed by the University of Warwick and the University of Edinburgh, and is jointly owned by NHS Health Scotland, the University of Warwick and the University of Edinburgh [29]. WEMWBS was developed to assess positive mental well-being in the general population and is widely used for the evaluation of projects, programs and policies which aim to improve mental well-being [29–31]. The WEMWB-scale measures aspects of both hedonic and eudaimonic well-being [30] and is a 14-item scale with 5 response categories. Individual scores are summed up and lead to one total score between 14 and 70 points. A higher total score indicates better mental well-being [29–31]. WEMWBS is an easy to use survey and has been used in previous studies with adults [32] and older adults [33], showing good validity and reliability.

### Movement behaviours

Movement behaviours (SB, LPA, and MVPA) and total sleep time (TST) were measured through accelerometry (Actigraph type wGT3X-BT, Actigraphcorp, Pensacola, FL, USA). The Actigraph wGT3X-BT is a wrist-worn accelerometer that measures and records physical movement associated with daily activity and sleep [34–36].

The Actigraph wGT3X-BT has been used in numerous studies to measure SB, PA, and sleep in older adults, and resulted in valid measurements for this target population [37–39]. In the present study, all participants were asked to wear the Actigraph device on their non-dominant wrist for a period of 6 consecutive days and five nights in which 2 weekend days were included. Accelerometer data were processed using well-established validated algorithms available in the Actilife software package (Actilife, v6.13.4) for wear time validation [35] and sleep/wake identification [40]. The following tri-axial vector magnitude (VM3) cut-points for wrist-worn actigraphy in older adults were used to identify sedentary time, LPA and MVPA [41, 42]. In line with prior research and regarding a minimal wear time, we only included data of participants with a minimum wear time of 4 wear days in total of at least 10 h of waking wear time data [35, 40, 43, 44].

### Data analysis

All analyses were conducted using R statistical systems (version 4.0.5, R Foundation for Statistical Computing, Vienna, Austria), using the packages Compositions and codaredistlm [45]. Physical behaviours were described as geometric means, normalized to represent minutes of each behaviour in a 1440-min day. Co-dependency of physical behaviours were described using a variation matrix [46]. Values closer to zero represent the higher co-dependency. The variance in the log ratio between factors ranged from 0.103 (total sleep time–sedentary time) to 0.550 (LPA–MVPA). There were no zero values, therefore no recoding was required. For an overview of the variation between the four movement behaviours, see Appendix 1.

Linear regression models were conducted using compositional data analysis procedures to examine the association of time spent in sleep, sedentary time, light-intensity physical activity (LPA) and moderate-to-vigorous intensity physical activity (MVPA) and mental well-being. Movement behaviours were expressed as isometric log-ratios (ILRs) (see Additional file 1 for an example formula and sign matrix) before being applied to the regression models as the independent variable. Briefly, ILRs were calculated using a sequential binary partition process and the sign matrix shown in Additional file 1. This ensures one part of the composite appears in the numerator of the first (ILR), with the other parts appearing in the denominator. Subsequent ILRs are then calculated by dropping the previous numerator, whilst recoding one of the denominators as the numerator. Model 1 was unadjusted, Model 2 was adjusted for age, sex, education, marital status and professional activity, and Model 3 additionally adjusted for smoking status and chronic conditions. β coefficients represent a 1-unit change in a given behaviour. In addition, the overall model fit was provided (R2).

Compositional isotemporal substitutions were conducted using similar methods as outlined previously [47–49]. Briefly, ILRs are calculated as described above, with time reallocate from one behaviour to another beforehand (see Additional files 1, 2). These ILRs are then applied to a linear model to show the association of the theoretical reallocation of time from one behaviour to another. These models examined the association of reallocating time from one behaviour to another. We focused on 30-min reallocations from SB to LPA, SB to MVPA, LPA to MVPA and SB to sleep time. Given the PA guidelines for older Adults [50], these reallocations are more favourable from a public health perspective, compared with for example reallocations from a higher intensity to lower intensity PA or SB [50]. In addition, although statistically modelled, 30-min reallocations are more realistic than longer time durations. Nevertheless, we did explore all possible reallocations between the four movement behaviours of 5 to 60 min at 5-min intervals. In short, isometric log-ratios were calculated for adjusted compositions (i.e., sedentary time − 30 min, MVPA + 30 min, sleep and LPA no change) and were then applied to multiple linear regression models as standard. Statistical significance was set at p ≤ 0.05.

## Results

Of the 453 adults participating in the study, 410 wore the accelerometer for 4 wear days of at least 10 h and were included in this analysis. The mean age of the 410 participants was 71.3 (SD 6.3) years, 71% were female and 77% were either married or lived together with a partner. Almost half (45%) had a low education level, but there was also good representation of participants with a medium (32%) or high educational level (20%). The majority (95%) was no longer professionally active. Just over half (54%) did not have a diagnosed chronic condition and three percent were current smokers (Table 1).

**Table 1:** Demographics and covariates

Characteristics of participants (n = 410)		WEMWBS score (mean, SD)
Age		
Mean, (SD)	71.3 (± 6.3)	53.75 (± 8.43)
Sample range	55.7–94.1	
Sex, n (%)		
Male	120 (29%)	54.74 (± 8.60)
Female	290 (71%)	53.35 (± 8.35)
Education^a^, n (%)		
Low	183 (45%)	51.50 (± 9.78)
Medium	133 (32%)	53.17 (± 8.18)
High	82 (20%)	54.78 (± 8.21)
Marital status, n (%)		
Married/cohabitant	315 (77%)	54.76 (± 7.53)
Single	95 (23%)	50.48 (± 10.24)
Professionally active, n (%)		
Yes	17 (4%)	53.65 (± 8.52)
No	389 (95%)	56.17 (± 6.99)
Smoking, n (%)		
Yes	14 (3%)	53.85 (± 8.46)
No	390 (95%)	53.50 (± 3.59)
Chronic condition, n (%)		
Yes	160 (39%)	54.20 (± 8.41)
No	222 (54%)	53.43 (± 8.71)
Missing	29 (7%)	

Only percentages of missing values > 5% were separately reported. Column percentages for the separate variables may therefore not equal 100%

^a^According to ISCED [28]

Overall, participants spent 23.6% or 339.4 min (5.66 h) of the 24-h day asleep (Table 2). The largest proportion of waking time was spent sedentary (57.8% of the 24-h day; 13.88 h), followed by smaller proportions of the 24-h day spent in LPA (10.73%; 2.58 h) and MVPA (7.83%; 1.89 h).

**Table 2:** Geometric means of physical behaviour composites

Behaviours	Hours/day	Percentage of 24-h
TST	5.65	23.57
SB	13.88	57.86
LPA	2.58	10.74
MVPA	1.89	7.83

*TST* total sleep time, *SB* sedentary behaviour, *LPA* light physical activity, *MVPA* moderate-to-vigorous physical activity

There were no statistically significant associations between time spent in the different behaviours and mental well-being, except for a positive association of LPA in the crude and partially adjusted models (Table 3). More specifically, more time spent in LPA, relative to time spent in the others behaviours, was associated with better well-being [Beta (SE) of 1.96 (0.99)] in the partially adjusted model. However, this association was no longer statistically significant in the fully adjusted model.

**Table 3:** Associations of SB, LPA, MVPA and sleep with well-being

Well-being	TST	SB	LPA	MVPA	Model fit
	Beta (SE)	Beta (SE)	Beta (SE)	Beta (SE)	p value
Model 1 Unadjusted	− 2.17 (1.60)	− 0.43 (1.71)	1.96 (0.98)*	0.64 (0.50)	0.169
Model 2 Partially adjusted	− 2.01 (1.68)	− 0.47 (1.87)	1.96 (0.99)*	0.52 (0.52)	**0.001**
Model 3 Fully adjusted	− 1.90 (1.77)	− 0.10 (1.99)	1.33 (1.07)	0.38 (0.56)	**0.010**

Values for each physical behaviour represent the association for time spent in each movement behaviour relative to all other behaviours [standard error (SE)]. β coefficients represent a 1-unit (hour) change in a given behaviour. Model 1 unadjusted. Model 2 partially adjusted for age, sex, education, marital status and professional activity. Model 3 fully adjusted for smoking status and chronic condition

*TST* total sleep time, *SB* sedentary behaviour, *LPA* light physical activity, *MVPA* moderate-to-vigorous physical activity

Significance levels: *p < 0.05. Significant results are also highlighted in bold

**Fig. 1:**
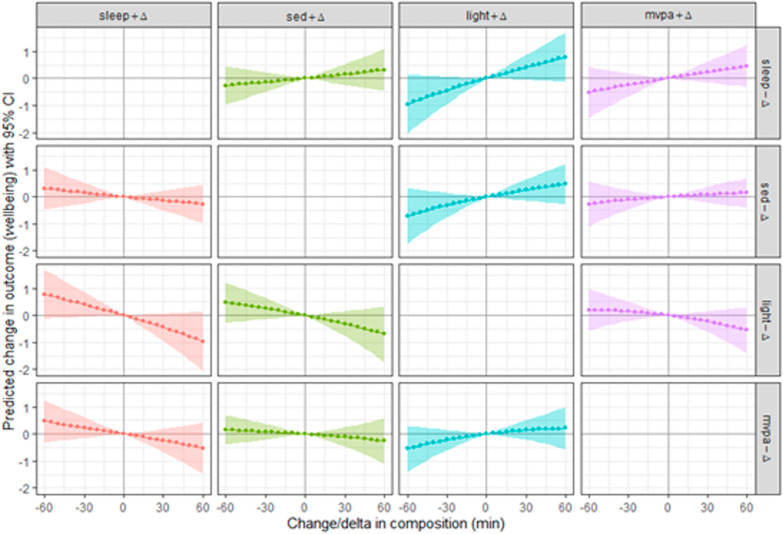
Graphical display of fully adjusted model 3

Reallocations of 30 min from SB to LPA, SB to MVPA, LPA to MVPA and SB to sleep time were not significantly associated with theoretical changes in mental well-being (Table 4). Changes in well-being for all theoretical substitutions of 5–60 min between the four movement behaviours are presented in Fig. 1 (fully adjusted model; for crude and partially adjusted models, see Appendixes 2 and 3). Most reallocations were not significantly associated with changes in well-being, but there were some statistically significant theoretical changes in well-being with substitutions larger than 30 min. However, the confidence intervals were approaching zero and the largest theoretical changes that were statistically significant were around two points on the 70-point WEMWBS, indicating that these changes were relatively small.

**Table 4:** Reallocations of 30 min from one physical behaviour to another

Well-being	SB to LPA		SB to MVPA		LPA to MVPA		SB to sleep	
	B	(95% CI)	B	(95% CI)	B	(95% CI)	B	(95% CI)
Model 1 Unadjusted	0.31	− 0.04; 0.67	0.14	− 0.11; 0.40	− 0.23	− 0.59; 0.12	− 0.15	− 0.46; 0.17
Model 2 Partially adjusted	0.32	− 0.05; 0.69	0.12	− 0.15; 0.39	− 0.26	− 0.62; 0.10	− 0.13	− 0.47; 0.21
Model 3 Fully adjusted	0.25	− 0.14; 0.65	0.08	− 0.21; 0.37	− 0.22	− 0.61; 0.16	− 0.14	− 0.49; 0.22

Model 1 unadjusted. Model 2 partially adjusted for age, sex, education, marital status and professional activity. Model 3 fully adjusted for smoking status and chronic condition

*TST* Total sleep time, *SB* sedentary behaviour, *LPA* light physical activity, *MVPA* moderate-to-vigorous physical activity

Significance levels: *p < 0.05

## Discussion

The aim of this study was twofold: (1) to describe the 24-h behaviours (light and moderate-vigorous physical activity, sedentary behaviour and sleep) and their associations with mental well-being in community dwelling adults aged 55+ years, and (2) to examine the theoretical changes in mental well-being if time were reallocated from one behaviour (TST, SB, LPA, MVPA) to another. To our knowledge, this is the first study to investigate the associations between physical activity, sedentary behaviour, sleep and mental well-being in adults aged 55+ years using objective measures of physical activity, sedentary behaviour and sleep (accelerometers) and composite measures of mental well-being (WEMWBS) to investigate any potential associations.

With respect to the first aim, the results showed significant positive associations between LPA and well-being, but only in the unadjusted and partially adjusted models. There were no significant associations between TST, SB, LPA or MVPA and well-being in the fully adjusted models. With respect to the second aim, we found no evidence of a significant association between the reallocation of up to 30 min of behaviours (TST, SB, LPA and MVPA) and mental well-being in adults aged 55+ years. Although there were some statistically significant changes in well-being with reallocations of more than 30 min, these were only small and not clinically relevant.

### Associations between isolated behaviours (TST, SB, LPA, MVPA) and well-being

Regarding associations between TST, SB, LPA or MVPA and well-being, this study could not report significant findings. However, based upon existing literature in younger populations, we would have expected to see associations of physical activity, sedentary behaviour and sleep with well-being in adults aged 55+ years.

Previous work has already described a negative relationship between physical inactivity and well-being in adults [10]; and a positive relationship between physical activity, sedentary behaviour and mental well-being in adults [10] and older adults [11, 12]. Furthermore, higher levels of sedentary behaviour were associated with lower levels of mental well-being in participants with lower levels of MVPA [12].

In line with the findings a study of Black et al. [51] examined the associations between device measured PA, mental well-being (WEMWBS) in adults aged 60–64 years and reported no statistically significant associations between PA and mental well-being. However, the results in this study of Black et al. [51] showed that participants who walked > 1 h/week had a 1.47 higher score on their WEMWBS scores when compared with participants who reported no walking. In this present study, we did not assess nor include walking as a separate variable in the analysis.

Based upon previous research, we would have expected to see significant associations between isolated behaviours (TST, SB, LPA and MVPA) and well-being in this study. Given that SB, PA or sleep as single factors were not significantly associated with well-being, in the next chapter we will elaborate on the behaviours (TST, SB, LPA and MVPA) within a 24-h continuum.

### Associations between the reallocation of behaviours within a 24-h continuum (TST, SB, LPA, MVPA) and mental well-being

Regarding isotemporal studies in the literature, no evidence has yet been found for time reallocations between behaviours (TST, SB, LPA, MVPA) within a 24-h continuum and well-being in adults aged 55+ years, although they have been found for other mental health outcomes, such as happiness [20]. In other populations by contrast, some studies did report significant associations of time reallocations: (1) between behaviours (PA, screen time and sleep) and well-being in adolescents [21]; and (2) between behaviours (TST, SB, LPA, MVPA) and well-being in adults [22]; and (3) between behaviours (TST, SB, LPA, MVPA) and well-being in in office workers [23].

A large scaled study of Brown and Kwan [21] examined cross-sectional associations of isotemporal substitutions between movement behaviours (MVPA) and mental well-being in adolescents (n = 1.118). Although this study included a younger population than is the case in our present study and although they only included subjective assessment tools, replacing 60 min of screen time by either MVPA or sleep, was associated with better mental well-being. Considering screen time as a form of sedentary behaviour, and if we assume that screen time equates to sedentary behaviour, this finding contradicts to what is presented in our present study that showed no association between reallocations of SB, PA or sleep and mental well-being. Although the time, position or context of screen time (as a form of sedentary behaviour) was not measured in this study, it could be so that different timing, positions and contexts could have showed different results in the associations with well-being.

In line with our present study, Brown and Kwan [21] found no significant associations with mental well-being when time (60 min) was reallocated of 60 min between MVPA and sleep.

Additionally, and in line with our findings, two studies on adults [22] and adult office workers [23] showed no significant associations between these behaviour compositions with the assessed mental health outcomes.

Although our present study did not show any significant associations with mental wellbeing when reallocating 30 min of time between behaviours (TST, SB, LPA and MVPA), there were some statistically significant theoretical changes in well-being with substitutions larger than 30 min. The lack of a higher number of significant associations of these 30 min reallocations in this study may be surprising given the previously observed positive associations of PA (as an isolated factor) and well-being in both adults [10] and older adults [11, 12]. Although behaviours (SB, PA and sleep) can be seen as isolated factors (aim 1), emerging research also looks at these behaviours within a 24-h continuum (aim 2) by means of isotemporal or compositional research [14]. Considering the statistical analyses are different (i.e. SB, PA and sleep as isolated factors in the analyses on the one hand and isotemporal or compositional analyses on the other hand), it is important to explore why these analyses might lead to different findings in the results.

Potential explanations for this lack of significant associations include (1) the measurement and conceptualization of SB, PA, and sleep, (2) the measurement and conceptualization of mental well-being and (3) the characteristics of the population in this study.

1. The measurement and conceptualization of SB, PA, and sleep
  This present study made use of device-measured data on SB, LPA, MVPA and sleep using Actigraph accelerometers (type wGT3X-BT). Accelerometry is considered the standard for objectively measuring SB providing valid estimates of SB [52]. Wrist-worn Actigraph accelerometers have also been widely used for measuring LPA and MVPA, and have shown to increase wear compliance in participants in free-living conditions [53–56]. In terms of sleep, the use of (non-dominant) wrist-worn accelerometers provides sleep data when people reside in their own natural environment, and has also been shown to be valid in older adults [55, 57–59].
  As PA levels tend to be overestimated when assessed by self-reported assessment tools [60], this could help explain why previous studies, using self-reported SB and PA, tend to show positive associations, whilst our study did not. A previous study of Domingos et al. [61] examined the associations between self-reported and device-measured PA in older adults and showed that self-reported versus objectively measured PA were associated differently with health outcomes such as depression, BMI and free fat mass. Although well-being was not included in this study of Domingos, based on the results of Domingos’ study, it is likely that self-reported and device measured PA are associated differently with well-being as well. Considering Actigraph accelerometers can provide valid measurements for SB, PA, and sleep in (older) adults [34–36] they are more likely to represent actual behaviours when compared to self-reported assessments with possible over- or underestimation of behaviours. This could explain why our present study showed different results when compared to studies that only made use of subjective measuring methods. Furthermore, given that each behaviour (TST, SB, LPA and MVPA) was seen as an isolated factor in the associations with well-being (approach 1), and later as a part of the 24-h continuum in the associations with well-being (approach 2), the other 24-h behaviours could have impacted the associations with well-being the latter approach.
2. The measurement and conceptualization of mental well-being
  Given that previous work made use of different scales to assess mental well-being, a comparison of mental well-being outcomes overall studies, is challenging. Mental well-being was assessed in previous studies by using scales such as WEMWBS [10–12, 51], the 12-item Short Form Health Survey (SF-12) [20], the Flourishing Scale, the Rosenberg Self-esteem scale and a brief resiliency scale of the Canadian Campus Well-being Survey [21], the Mental-health related QoL [22] and The Hospital Anxiety and Depression Scale [23]. Furthermore, happiness was also measured in one study by means of the Cantril Ladder of Life Scale [20]. In this present work we chose to use WEMWBS as it is a valid survey to assess positive mental well-being [29–31]. Previous studies that reported on associations of time reallocation between behaviours with mental well-being did however not report the use of WEMWBS [21–23]. Comparison between the findings in our studies and previous work is therefore imprecise.
3. The characteristics of the population in this study
  The study population in this study was recruited from one single socio-cultural organization that showed to be more physically active and less sedentary compared with the general Belgian population [62]. Also, the adults aged 55+ years in this study reported a higher mean WEMWBS score (53.8), when compared with the mean demographic scores for adults aged 55+ years (51.4), older adults aged 65+ years (52.4) and older adults aged 75+ years (51.2) [29, 30, 32].
  In line with prior discussed research of Harvey et al. [10] this study found that physically inactive participants showed lower WEMWBS scores when compared to physically active adults. Given this rather physically active study population, this study population could be less likely to benefit from increasing PA and therefore mental well-being.
  Additionally, this sample consisted of 71% females. Previous research of Matud et al. [62] examined the relevance of gender to well-being in adults. This study concluded that male adults scored better well-being than females in terms of self-acceptance and autonomy, while female adults scored higher than males in personal growth and positive relations with others [62, 63]. In this present study, the mean WEMWBS scores between males and females were not significantly different. Although we did control for gender in our analyses in the adjusted model (model 2), we did not divide the study population in two subgroups of males and females.
  Almost half of the participants in this study (45%) indicated that elementary education was their highest achieved form of education, which was categorized as low educated [28] . Based on previous work of Sheikh et al. [64] low education is associated with a lower socio-economic status, which in turn is associated with being unhealthy and having a low level of well-being. Although 54% in this study population indicated that they did not suffer from (one or more) chronic conditions, this generally healthy study population could have concealed the impact of a lower educational status and a lower well-being.

### Strengths and limitations

This study has several strengths and limitations. Strengths include the large sample size in adults aged 55+ years and the combined use of device-based measures of physical activity, sedentary behaviour and sleep data with subjective data from WEMWBS, measuring both eudaimonic and hedonic well-being.

Limitations include the cross-sectional design and the generalizability of the findings. First, in this cross-sectional design, PA, SB and sleep outcomes were measured at the same time and the analyses were based on statistical modelling (Coda and ISM) rather than on real-life changes in SB, LPA, MVPA and sleep behaviours. Although we examined the associations of these 24-h behaviours with mental well-being, we cannot exclude the possibility of bi-directional associations. Second, as described in the previous section and potentially as result of selection bias, this sample was not representative for the general Flemish population aged 55+ years in terms of well-being, physical activity level, proportion of males/females, education and general health. Given the included sample, the conclusions in this study apply to generally healthy and physically active older adults rather than to older adults with specific chronic conditions. The analysis only examined ‘one to one’ reallocations, meaning it was not possible to determine the estimated impact on wellbeing if time spent in one behaviour was reallocated to all other behaviours equally. Future research should look to assess this question.

## Conclusion

Based on the analyses in this study, this study showed a significant positive association between LPA and well-being, but only in the unadjusted and partially adjusted models. There were no significant associations between TST, SB, LPA or MVPA and well-being in the fully adjusted models. Furthermore, we found no evidence of a significant association between the reallocation of behaviours (TST, SB, LPA and MVPA) and mental well-being in adults aged 55+ years. Considering well-being as a key aspect for healthy ageing, future public health research should continue to examine physical activity (both light and moderate-to-vigorous), sedentary behaviour, as well as sleep as a part of the 24-h continuum and its associations with mental health outcomes in older adults.

## Supplementary Information

**Additional file 1.** Example sign matrix and formulas.

**Additional file 2.** Results of all behavioural reallocations.

## Data Availability

The datasets used and/or analysed during the current study are available from the corresponding author on reasonable request.

## Appendix 1: Variation matrix

**Table:**

Behaviours	Total sleep time	Sedentary time	LPA	MVPA
TST	–	0.103	0.175	0.315
SB	0.103	–	0.121	0.187
LPA	0.175	0.121	–	0.550
MVPA	0.315	0.187	0.550	–

Data present the variance in the log ratio between factors. Numbers closer to zero represent factors that have a high level of codependency

*TST* total sleep time, *SB* sedentary behaviour, *LPA* light physical activity, *MVPA* moderate-to-vigorous physical activity

## Abbreviations

ISCED: International Standard Classification of Education
LPA: Light physical activity
Min: Minutes
MVPA: Moderate to vigorous activity
PA: Physical activity
SB: Sedentary behaviour
TST: Total sleep time
VM: Vector magnitude
WEMWBS: Warwick-Edinburgh Mental Well-being Scale
WHO: World Health Organization
