# Supplementary material for: To be well or not to be well: compositional associations of physical activity, sedentary behaviour and sleep with mental well-being in Flemish adults aged 55+ years
Source: J Act Sedentary Sleep Behav. 2023 May 1;2:9. doi: 10.1186/s44167-023-00019-3 (PMC11960346; doi:10.1186/s44167-023-00019-3)
Supplement: Supplementary file 1 — Additional file 1. Example sign matrix and formulas. [file 44167_2023_19_MOESM1_ESM.docx]

**Example sign matrix**

|  | Sleep | Sedentary | LPA | MVPA |
| --- | --- | --- | --- | --- |
| Ilr1 | 1 | -1 | -1 | -1 |
| Ilr2 | 0 | 1 | -1 | -1 |
| Ilr3 | 0 | 0 | 1 | -1 |

**Example formulas for isometric log ratios for linear regression**

$$ilr1=ln\left( \frac{\sqrt{sleep}}{\sqrt{sedentary+LPA+MVPA}} \right)$$

$$\mathrm{ilr}2=ln\left( \frac{\sqrt{sedentary}}{\sqrt{LPA+MVPA}} \right)$$

$$\mathrm{ilr}3=ln\left( \frac{\sqrt{LPA}}{\sqrt{MVPA}} \right)$$

**Example formulas for isometric log ratios for compositional isotemporal substitution**

$$ilr1=\sqrt{\frac{3}{4}}*ln\left( \frac{\sqrt{sleep}}{\sqrt{sedentary+LPA+{MVPA}^{1/3}}} \right)$$

$$\mathrm{ilr}2=\sqrt{\frac{2}{3}}*ln\left( \frac{\sqrt{sedentary}}{\sqrt{LPA+{MVPA}^{1/2}}} \right)$$

$$\mathrm{ilr}3=\sqrt{\frac{1}{2}}*ln\left( \frac{\sqrt{LPA}}{\sqrt{MVPA}} \right)$$
