# Supplementary material for: To be well or not to be well: compositional associations of physical activity, sedentary behaviour and sleep with mental well-being in Flemish adults aged 55+ years
Source: J Act Sedentary Sleep Behav. 2023 May 1;2:9. doi: 10.1186/s44167-023-00019-3 (PMC11960346; doi:10.1186/s44167-023-00019-3)
Supplement: Supplementary file 2 — Additional file 2. Results of all behavioural reallocations. [file 44167_2023_19_MOESM2_ESM.docx]

**Results of all behavioural reallocations**

**Model 1**

| **Time** | **comp+** | **comp-** | **delta_pred** | **ci_lo** | **ci_up** |
| --- | --- | --- | --- | --- | --- |
| Minus60 | sleep | sed | 0.34 | -0.35 | 1.03 |
| Minus60 | sleep | light | 0.92 | 0.07 | 1.77 |
| Minus60 | sleep | mvpa | 0.60 | -0.11 | 1.31 |
| Minus60 | sed | sleep | -0.28 | -0.90 | 0.34 |
| Minus60 | sed | light | 0.58 | -0.09 | 1.25 |
| Minus60 | sed | mvpa | 0.26 | -0.21 | 0.73 |
| Minus60 | light | sleep | -1.14 | -2.16 | -0.11 |
| Minus60 | light | sed | -0.86 | -1.78 | 0.07 |
| Minus60 | light | mvpa | -0.60 | -1.39 | 0.19 |
| Minus60 | mvpa | sleep | -0.73 | -1.60 | 0.14 |
| Minus60 | mvpa | sed | -0.45 | -1.17 | 0.28 |
| Minus60 | mvpa | light | 0.14 | -0.58 | 0.85 |
| Minus55 | sleep | sed | 0.31 | -0.32 | 0.94 |
| Minus55 | sleep | light | 0.85 | 0.07 | 1.63 |
| Minus55 | sleep | mvpa | 0.55 | -0.10 | 1.20 |
| Minus55 | sed | sleep | -0.26 | -0.83 | 0.31 |
| Minus55 | sed | light | 0.54 | -0.08 | 1.16 |
| Minus55 | sed | mvpa | 0.25 | -0.19 | 0.68 |
| Minus55 | light | sleep | -1.03 | -1.95 | -0.10 |
| Minus55 | light | sed | -0.77 | -1.60 | 0.06 |
| Minus55 | light | mvpa | -0.52 | -1.23 | 0.18 |
| Minus55 | mvpa | sleep | -0.65 | -1.43 | 0.13 |
| Minus55 | mvpa | sed | -0.39 | -1.04 | 0.25 |
| Minus55 | mvpa | light | 0.15 | -0.50 | 0.79 |
| Minus50 | sleep | sed | 0.28 | -0.29 | 0.85 |
| Minus50 | sleep | light | 0.77 | 0.06 | 1.49 |
| Minus50 | sleep | mvpa | 0.50 | -0.09 | 1.09 |
| Minus50 | sed | sleep | -0.24 | -0.75 | 0.28 |
| Minus50 | sed | light | 0.50 | -0.07 | 1.07 |
| Minus50 | sed | mvpa | 0.23 | -0.17 | 0.63 |
| Minus50 | light | sleep | -0.92 | -1.75 | -0.09 |
| Minus50 | light | sed | -0.68 | -1.42 | 0.06 |
| Minus50 | light | mvpa | -0.46 | -1.09 | 0.17 |
| Minus50 | mvpa | sleep | -0.58 | -1.28 | 0.11 |
| Minus50 | mvpa | sed | -0.35 | -0.91 | 0.22 |
| Minus50 | mvpa | light | 0.15 | -0.42 | 0.72 |
| Minus45 | sleep | sed | 0.25 | -0.26 | 0.76 |
| Minus45 | sleep | light | 0.70 | 0.06 | 1.34 |
| Minus45 | sleep | mvpa | 0.45 | -0.08 | 0.99 |
| Minus45 | sed | sleep | -0.21 | -0.68 | 0.26 |
| Minus45 | sed | light | 0.45 | -0.06 | 0.97 |
| Minus45 | sed | mvpa | 0.21 | -0.16 | 0.57 |
| Minus45 | light | sleep | -0.82 | -1.55 | -0.08 |
| Minus45 | light | sed | -0.60 | -1.26 | 0.05 |
| Minus45 | light | mvpa | -0.40 | -0.96 | 0.16 |
| Minus45 | mvpa | sleep | -0.52 | -1.13 | 0.10 |
| Minus45 | mvpa | sed | -0.30 | -0.80 | 0.20 |
| Minus45 | mvpa | light | 0.15 | -0.36 | 0.66 |
| Minus40 | sleep | sed | 0.22 | -0.23 | 0.67 |
| Minus40 | sleep | light | 0.63 | 0.05 | 1.20 |
| Minus40 | sleep | mvpa | 0.40 | -0.07 | 0.88 |
| Minus40 | sed | sleep | -0.19 | -0.61 | 0.23 |
| Minus40 | sed | light | 0.41 | -0.06 | 0.87 |
| Minus40 | sed | mvpa | 0.19 | -0.14 | 0.51 |
| Minus40 | light | sleep | -0.72 | -1.36 | -0.07 |
| Minus40 | light | sed | -0.52 | -1.10 | 0.05 |
| Minus40 | light | mvpa | -0.34 | -0.83 | 0.15 |
| Minus40 | mvpa | sleep | -0.45 | -0.99 | 0.08 |
| Minus40 | mvpa | sed | -0.26 | -0.69 | 0.17 |
| Minus40 | mvpa | light | 0.15 | -0.30 | 0.60 |
| Minus35 | sleep | sed | 0.19 | -0.20 | 0.58 |
| Minus35 | sleep | light | 0.55 | 0.05 | 1.05 |
| Minus35 | sleep | mvpa | 0.35 | -0.06 | 0.77 |
| Minus35 | sed | sleep | -0.17 | -0.54 | 0.20 |
| Minus35 | sed | light | 0.36 | -0.05 | 0.77 |
| Minus35 | sed | mvpa | 0.17 | -0.12 | 0.45 |
| Minus35 | light | sleep | -0.62 | -1.18 | -0.06 |
| Minus35 | light | sed | -0.45 | -0.94 | 0.04 |
| Minus35 | light | mvpa | -0.29 | -0.71 | 0.14 |
| Minus35 | mvpa | sleep | -0.39 | -0.85 | 0.07 |
| Minus35 | mvpa | sed | -0.22 | -0.59 | 0.15 |
| Minus35 | mvpa | light | 0.14 | -0.25 | 0.53 |
| Minus30 | sleep | sed | 0.16 | -0.17 | 0.49 |
| Minus30 | sleep | light | 0.47 | 0.04 | 0.91 |
| Minus30 | sleep | mvpa | 0.30 | -0.05 | 0.66 |
| Minus30 | sed | sleep | -0.15 | -0.46 | 0.17 |
| Minus30 | sed | light | 0.31 | -0.04 | 0.67 |
| Minus30 | sed | mvpa | 0.14 | -0.11 | 0.40 |
| Minus30 | light | sleep | -0.52 | -1.00 | -0.05 |
| Minus30 | light | sed | -0.38 | -0.79 | 0.04 |
| Minus30 | light | mvpa | -0.23 | -0.59 | 0.12 |
| Minus30 | mvpa | sleep | -0.33 | -0.72 | 0.06 |
| Minus30 | mvpa | sed | -0.18 | -0.49 | 0.12 |
| Minus30 | mvpa | light | 0.13 | -0.20 | 0.46 |
| Minus25 | sleep | sed | 0.13 | -0.14 | 0.41 |
| Minus25 | sleep | light | 0.40 | 0.03 | 0.76 |
| Minus25 | sleep | mvpa | 0.25 | -0.04 | 0.55 |
| Minus25 | sed | sleep | -0.12 | -0.39 | 0.14 |
| Minus25 | sed | light | 0.27 | -0.03 | 0.56 |
| Minus25 | sed | mvpa | 0.12 | -0.09 | 0.33 |
| Minus25 | light | sleep | -0.43 | -0.82 | -0.04 |
| Minus25 | light | sed | -0.31 | -0.65 | 0.03 |
| Minus25 | light | mvpa | -0.19 | -0.48 | 0.10 |
| Minus25 | mvpa | sleep | -0.27 | -0.59 | 0.05 |
| Minus25 | mvpa | sed | -0.15 | -0.40 | 0.10 |
| Minus25 | mvpa | light | 0.12 | -0.16 | 0.39 |
| Minus20 | sleep | sed | 0.11 | -0.12 | 0.33 |
| Minus20 | sleep | light | 0.32 | 0.03 | 0.61 |
| Minus20 | sleep | mvpa | 0.20 | -0.04 | 0.44 |
| Minus20 | sed | sleep | -0.10 | -0.31 | 0.11 |
| Minus20 | sed | light | 0.22 | -0.03 | 0.46 |
| Minus20 | sed | mvpa | 0.10 | -0.07 | 0.27 |
| Minus20 | light | sleep | -0.34 | -0.65 | -0.03 |
| Minus20 | light | sed | -0.24 | -0.51 | 0.02 |
| Minus20 | light | mvpa | -0.14 | -0.38 | 0.09 |
| Minus20 | mvpa | sleep | -0.22 | -0.47 | 0.04 |
| Minus20 | mvpa | sed | -0.12 | -0.31 | 0.08 |
| Minus20 | mvpa | light | 0.10 | -0.12 | 0.32 |
| Minus15 | sleep | sed | 0.08 | -0.09 | 0.24 |
| Minus15 | sleep | light | 0.24 | 0.02 | 0.46 |
| Minus15 | sleep | mvpa | 0.15 | -0.03 | 0.34 |
| Minus15 | sed | sleep | -0.07 | -0.23 | 0.09 |
| Minus15 | sed | light | 0.16 | -0.02 | 0.35 |
| Minus15 | sed | mvpa | 0.08 | -0.05 | 0.21 |
| Minus15 | light | sleep | -0.25 | -0.48 | -0.02 |
| Minus15 | light | sed | -0.18 | -0.38 | 0.02 |
| Minus15 | light | mvpa | -0.10 | -0.28 | 0.07 |
| Minus15 | mvpa | sleep | -0.16 | -0.35 | 0.03 |
| Minus15 | mvpa | sed | -0.09 | -0.23 | 0.06 |
| Minus15 | mvpa | light | 0.08 | -0.09 | 0.24 |
| Minus10 | sleep | sed | 0.05 | -0.06 | 0.16 |
| Minus10 | sleep | light | 0.16 | 0.01 | 0.31 |
| Minus10 | sleep | mvpa | 0.10 | -0.02 | 0.22 |
| Minus10 | sed | sleep | -0.05 | -0.16 | 0.06 |
| Minus10 | sed | light | 0.11 | -0.01 | 0.23 |
| Minus10 | sed | mvpa | 0.05 | -0.04 | 0.14 |
| Minus10 | light | sleep | -0.17 | -0.32 | -0.02 |
| Minus10 | light | sed | -0.12 | -0.25 | 0.01 |
| Minus10 | light | mvpa | -0.07 | -0.18 | 0.05 |
| Minus10 | mvpa | sleep | -0.11 | -0.23 | 0.02 |
| Minus10 | mvpa | sed | -0.06 | -0.15 | 0.04 |
| Minus10 | mvpa | light | 0.05 | -0.06 | 0.17 |
| Minus5 | sleep | sed | 0.03 | -0.03 | 0.08 |
| Minus5 | sleep | light | 0.08 | 0.01 | 0.16 |
| Minus5 | sleep | mvpa | 0.05 | -0.01 | 0.11 |
| Minus5 | sed | sleep | -0.03 | -0.08 | 0.03 |
| Minus5 | sed | light | 0.06 | -0.01 | 0.12 |
| Minus5 | sed | mvpa | 0.03 | -0.02 | 0.07 |
| Minus5 | light | sleep | -0.08 | -0.16 | -0.01 |
| Minus5 | light | sed | -0.06 | -0.12 | 0.01 |
| Minus5 | light | mvpa | -0.03 | -0.09 | 0.02 |
| Minus5 | mvpa | sleep | -0.05 | -0.11 | 0.01 |
| Minus5 | mvpa | sed | -0.03 | -0.07 | 0.02 |
| Minus5 | mvpa | light | 0.03 | -0.03 | 0.08 |
| Plus5 | sleep | sed | -0.03 | -0.08 | 0.03 |
| Plus5 | sleep | light | -0.08 | -0.16 | -0.01 |
| Plus5 | sleep | mvpa | -0.05 | -0.11 | 0.01 |
| Plus5 | sed | sleep | 0.03 | -0.03 | 0.08 |
| Plus5 | sed | light | -0.06 | -0.12 | 0.01 |
| Plus5 | sed | mvpa | -0.03 | -0.07 | 0.02 |
| Plus5 | light | sleep | 0.08 | 0.01 | 0.16 |
| Plus5 | light | sed | 0.06 | -0.01 | 0.12 |
| Plus5 | light | mvpa | 0.03 | -0.03 | 0.08 |
| Plus5 | mvpa | sleep | 0.05 | -0.01 | 0.11 |
| Plus5 | mvpa | sed | 0.03 | -0.02 | 0.07 |
| Plus5 | mvpa | light | -0.03 | -0.09 | 0.02 |
| Plus10 | sleep | sed | -0.05 | -0.16 | 0.06 |
| Plus10 | sleep | light | -0.17 | -0.32 | -0.02 |
| Plus10 | sleep | mvpa | -0.11 | -0.23 | 0.02 |
| Plus10 | sed | sleep | 0.05 | -0.06 | 0.16 |
| Plus10 | sed | light | -0.12 | -0.25 | 0.01 |
| Plus10 | sed | mvpa | -0.06 | -0.15 | 0.04 |
| Plus10 | light | sleep | 0.16 | 0.01 | 0.31 |
| Plus10 | light | sed | 0.11 | -0.01 | 0.23 |
| Plus10 | light | mvpa | 0.05 | -0.06 | 0.17 |
| Plus10 | mvpa | sleep | 0.10 | -0.02 | 0.22 |
| Plus10 | mvpa | sed | 0.05 | -0.04 | 0.14 |
| Plus10 | mvpa | light | -0.07 | -0.18 | 0.05 |
| Plus15 | sleep | sed | -0.07 | -0.23 | 0.09 |
| Plus15 | sleep | light | -0.25 | -0.48 | -0.02 |
| Plus15 | sleep | mvpa | -0.16 | -0.35 | 0.03 |
| Plus15 | sed | sleep | 0.08 | -0.09 | 0.24 |
| Plus15 | sed | light | -0.18 | -0.38 | 0.02 |
| Plus15 | sed | mvpa | -0.09 | -0.23 | 0.06 |
| Plus15 | light | sleep | 0.24 | 0.02 | 0.46 |
| Plus15 | light | sed | 0.16 | -0.02 | 0.35 |
| Plus15 | light | mvpa | 0.08 | -0.09 | 0.24 |
| Plus15 | mvpa | sleep | 0.15 | -0.03 | 0.34 |
| Plus15 | mvpa | sed | 0.08 | -0.05 | 0.21 |
| Plus15 | mvpa | light | -0.10 | -0.28 | 0.07 |
| Plus20 | sleep | sed | -0.10 | -0.31 | 0.11 |
| Plus20 | sleep | light | -0.34 | -0.65 | -0.03 |
| Plus20 | sleep | mvpa | -0.22 | -0.47 | 0.04 |
| Plus20 | sed | sleep | 0.11 | -0.12 | 0.33 |
| Plus20 | sed | light | -0.24 | -0.51 | 0.02 |
| Plus20 | sed | mvpa | -0.12 | -0.31 | 0.08 |
| Plus20 | light | sleep | 0.32 | 0.03 | 0.61 |
| Plus20 | light | sed | 0.22 | -0.03 | 0.46 |
| Plus20 | light | mvpa | 0.10 | -0.12 | 0.32 |
| Plus20 | mvpa | sleep | 0.20 | -0.04 | 0.44 |
| Plus20 | mvpa | sed | 0.10 | -0.07 | 0.27 |
| Plus20 | mvpa | light | -0.14 | -0.38 | 0.09 |
| Plus25 | sleep | sed | -0.12 | -0.39 | 0.14 |
| Plus25 | sleep | light | -0.43 | -0.82 | -0.04 |
| Plus25 | sleep | mvpa | -0.27 | -0.59 | 0.05 |
| Plus25 | sed | sleep | 0.13 | -0.14 | 0.41 |
| Plus25 | sed | light | -0.31 | -0.65 | 0.03 |
| Plus25 | sed | mvpa | -0.15 | -0.40 | 0.10 |
| Plus25 | light | sleep | 0.40 | 0.03 | 0.76 |
| Plus25 | light | sed | 0.27 | -0.03 | 0.56 |
| Plus25 | light | mvpa | 0.12 | -0.16 | 0.39 |
| Plus25 | mvpa | sleep | 0.25 | -0.04 | 0.55 |
| Plus25 | mvpa | sed | 0.12 | -0.09 | 0.33 |
| Plus25 | mvpa | light | -0.19 | -0.48 | 0.10 |
| Plus30 | sleep | sed | -0.15 | -0.46 | 0.17 |
| Plus30 | sleep | light | -0.52 | -1.00 | -0.05 |
| Plus30 | sleep | mvpa | -0.33 | -0.72 | 0.06 |
| Plus30 | sed | sleep | 0.16 | -0.17 | 0.49 |
| Plus30 | sed | light | -0.38 | -0.79 | 0.04 |
| Plus30 | sed | mvpa | -0.18 | -0.49 | 0.12 |
| Plus30 | light | sleep | 0.47 | 0.04 | 0.91 |
| Plus30 | light | sed | 0.31 | -0.04 | 0.67 |
| Plus30 | light | mvpa | 0.13 | -0.20 | 0.46 |
| Plus30 | mvpa | sleep | 0.30 | -0.05 | 0.66 |
| Plus30 | mvpa | sed | 0.14 | -0.11 | 0.40 |
| Plus30 | mvpa | light | -0.23 | -0.59 | 0.12 |
| Plus35 | sleep | sed | -0.17 | -0.54 | 0.20 |
| Plus35 | sleep | light | -0.62 | -1.18 | -0.06 |
| Plus35 | sleep | mvpa | -0.39 | -0.85 | 0.07 |
| Plus35 | sed | sleep | 0.19 | -0.20 | 0.58 |
| Plus35 | sed | light | -0.45 | -0.94 | 0.04 |
| Plus35 | sed | mvpa | -0.22 | -0.59 | 0.15 |
| Plus35 | light | sleep | 0.55 | 0.05 | 1.05 |
| Plus35 | light | sed | 0.36 | -0.05 | 0.77 |
| Plus35 | light | mvpa | 0.14 | -0.25 | 0.53 |
| Plus35 | mvpa | sleep | 0.35 | -0.06 | 0.77 |
| Plus35 | mvpa | sed | 0.17 | -0.12 | 0.45 |
| Plus35 | mvpa | light | -0.29 | -0.71 | 0.14 |
| Plus40 | sleep | sed | -0.19 | -0.61 | 0.23 |
| Plus40 | sleep | light | -0.72 | -1.36 | -0.07 |
| Plus40 | sleep | mvpa | -0.45 | -0.99 | 0.08 |
| Plus40 | sed | sleep | 0.22 | -0.23 | 0.67 |
| Plus40 | sed | light | -0.52 | -1.10 | 0.05 |
| Plus40 | sed | mvpa | -0.26 | -0.69 | 0.17 |
| Plus40 | light | sleep | 0.63 | 0.05 | 1.20 |
| Plus40 | light | sed | 0.41 | -0.06 | 0.87 |
| Plus40 | light | mvpa | 0.15 | -0.30 | 0.60 |
| Plus40 | mvpa | sleep | 0.40 | -0.07 | 0.88 |
| Plus40 | mvpa | sed | 0.19 | -0.14 | 0.51 |
| Plus40 | mvpa | light | -0.34 | -0.83 | 0.15 |
| Plus45 | sleep | sed | -0.21 | -0.68 | 0.26 |
| Plus45 | sleep | light | -0.82 | -1.55 | -0.08 |
| Plus45 | sleep | mvpa | -0.52 | -1.13 | 0.10 |
| Plus45 | sed | sleep | 0.25 | -0.26 | 0.76 |
| Plus45 | sed | light | -0.60 | -1.26 | 0.05 |
| Plus45 | sed | mvpa | -0.30 | -0.80 | 0.20 |
| Plus45 | light | sleep | 0.70 | 0.06 | 1.34 |
| Plus45 | light | sed | 0.45 | -0.06 | 0.97 |
| Plus45 | light | mvpa | 0.15 | -0.36 | 0.66 |
| Plus45 | mvpa | sleep | 0.45 | -0.08 | 0.99 |
| Plus45 | mvpa | sed | 0.21 | -0.16 | 0.57 |
| Plus45 | mvpa | light | -0.40 | -0.96 | 0.16 |
| Plus50 | sleep | sed | -0.24 | -0.75 | 0.28 |
| Plus50 | sleep | light | -0.92 | -1.75 | -0.09 |
| Plus50 | sleep | mvpa | -0.58 | -1.28 | 0.11 |
| Plus50 | sed | sleep | 0.28 | -0.29 | 0.85 |
| Plus50 | sed | light | -0.68 | -1.42 | 0.06 |
| Plus50 | sed | mvpa | -0.35 | -0.91 | 0.22 |
| Plus50 | light | sleep | 0.77 | 0.06 | 1.49 |
| Plus50 | light | sed | 0.50 | -0.07 | 1.07 |
| Plus50 | light | mvpa | 0.15 | -0.42 | 0.72 |
| Plus50 | mvpa | sleep | 0.50 | -0.09 | 1.09 |
| Plus50 | mvpa | sed | 0.23 | -0.17 | 0.63 |
| Plus50 | mvpa | light | -0.46 | -1.09 | 0.17 |
| Plus55 | sleep | sed | -0.26 | -0.83 | 0.31 |
| Plus55 | sleep | light | -1.03 | -1.95 | -0.10 |
| Plus55 | sleep | mvpa | -0.65 | -1.43 | 0.13 |
| Plus55 | sed | sleep | 0.31 | -0.32 | 0.94 |
| Plus55 | sed | light | -0.77 | -1.60 | 0.06 |
| Plus55 | sed | mvpa | -0.39 | -1.04 | 0.25 |
| Plus55 | light | sleep | 0.85 | 0.07 | 1.63 |
| Plus55 | light | sed | 0.54 | -0.08 | 1.16 |
| Plus55 | light | mvpa | 0.15 | -0.50 | 0.79 |
| Plus55 | mvpa | sleep | 0.55 | -0.10 | 1.20 |
| Plus55 | mvpa | sed | 0.25 | -0.19 | 0.68 |
| Plus55 | mvpa | light | -0.52 | -1.23 | 0.18 |
| Plus60 | sleep | sed | -0.28 | -0.90 | 0.34 |
| Plus60 | sleep | light | -1.14 | -2.16 | -0.11 |
| Plus60 | sleep | mvpa | -0.73 | -1.60 | 0.14 |
| Plus60 | sed | sleep | 0.34 | -0.35 | 1.03 |
| Plus60 | sed | light | -0.86 | -1.78 | 0.07 |
| Plus60 | sed | mvpa | -0.45 | -1.17 | 0.28 |
| Plus60 | light | sleep | 0.92 | 0.07 | 1.77 |
| Plus60 | light | sed | 0.58 | -0.09 | 1.25 |
| Plus60 | light | mvpa | 0.14 | -0.58 | 0.85 |
| Plus60 | mvpa | sleep | 0.60 | -0.11 | 1.31 |
| Plus60 | mvpa | sed | 0.26 | -0.21 | 0.73 |
| Plus60 | mvpa | light | -0.60 | -1.39 | 0.19 |

**Model 2**

| **Time** | **comp+** | **comp-** | **delta_pred** | **ci_lo** | **ci_up** |
| --- | --- | --- | --- | --- | --- |
| Minus60 | sleep | sed | 0.31 | -0.43 | 1.05 |
| Minus60 | sleep | light | 0.90 | 0.04 | 1.75 |
| Minus60 | sleep | mvpa | 0.53 | -0.19 | 1.25 |
| Minus60 | sed | sleep | -0.25 | -0.92 | 0.41 |
| Minus60 | sed | light | 0.59 | -0.11 | 1.29 |
| Minus60 | sed | mvpa | 0.22 | -0.29 | 0.73 |
| Minus60 | light | sleep | -1.12 | -2.15 | -0.10 |
| Minus60 | light | sed | -0.87 | -1.82 | 0.09 |
| Minus60 | light | mvpa | -0.65 | -1.45 | 0.15 |
| Minus60 | mvpa | sleep | -0.63 | -1.51 | 0.26 |
| Minus60 | mvpa | sed | -0.37 | -1.15 | 0.41 |
| Minus60 | mvpa | light | 0.22 | -0.52 | 0.95 |
| Minus55 | sleep | sed | 0.28 | -0.39 | 0.95 |
| Minus55 | sleep | light | 0.83 | 0.04 | 1.61 |
| Minus55 | sleep | mvpa | 0.49 | -0.18 | 1.15 |
| Minus55 | sed | sleep | -0.23 | -0.84 | 0.38 |
| Minus55 | sed | light | 0.55 | -0.10 | 1.19 |
| Minus55 | sed | mvpa | 0.21 | -0.26 | 0.68 |
| Minus55 | light | sleep | -1.01 | -1.94 | -0.09 |
| Minus55 | light | sed | -0.78 | -1.63 | 0.08 |
| Minus55 | light | mvpa | -0.57 | -1.29 | 0.15 |
| Minus55 | mvpa | sleep | -0.56 | -1.36 | 0.23 |
| Minus55 | mvpa | sed | -0.33 | -1.02 | 0.37 |
| Minus55 | mvpa | light | 0.22 | -0.44 | 0.88 |
| Minus50 | sleep | sed | 0.25 | -0.35 | 0.86 |
| Minus50 | sleep | light | 0.76 | 0.04 | 1.47 |
| Minus50 | sleep | mvpa | 0.44 | -0.16 | 1.05 |
| Minus50 | sed | sleep | -0.21 | -0.77 | 0.34 |
| Minus50 | sed | light | 0.50 | -0.09 | 1.09 |
| Minus50 | sed | mvpa | 0.19 | -0.24 | 0.62 |
| Minus50 | light | sleep | -0.91 | -1.74 | -0.08 |
| Minus50 | light | sed | -0.69 | -1.45 | 0.07 |
| Minus50 | light | mvpa | -0.50 | -1.14 | 0.14 |
| Minus50 | mvpa | sleep | -0.50 | -1.21 | 0.20 |
| Minus50 | mvpa | sed | -0.29 | -0.90 | 0.32 |
| Minus50 | mvpa | light | 0.21 | -0.38 | 0.80 |
| Minus45 | sleep | sed | 0.23 | -0.32 | 0.77 |
| Minus45 | sleep | light | 0.68 | 0.04 | 1.33 |
| Minus45 | sleep | mvpa | 0.40 | -0.14 | 0.94 |
| Minus45 | sed | sleep | -0.19 | -0.70 | 0.31 |
| Minus45 | sed | light | 0.46 | -0.08 | 0.99 |
| Minus45 | sed | mvpa | 0.17 | -0.22 | 0.57 |
| Minus45 | light | sleep | -0.80 | -1.54 | -0.07 |
| Minus45 | light | sed | -0.61 | -1.28 | 0.07 |
| Minus45 | light | mvpa | -0.44 | -1.00 | 0.13 |
| Minus45 | mvpa | sleep | -0.45 | -1.07 | 0.18 |
| Minus45 | mvpa | sed | -0.25 | -0.79 | 0.28 |
| Minus45 | mvpa | light | 0.21 | -0.32 | 0.73 |
| Minus40 | sleep | sed | 0.20 | -0.28 | 0.68 |
| Minus40 | sleep | light | 0.61 | 0.03 | 1.19 |
| Minus40 | sleep | mvpa | 0.35 | -0.13 | 0.84 |
| Minus40 | sed | sleep | -0.17 | -0.62 | 0.27 |
| Minus40 | sed | light | 0.41 | -0.07 | 0.89 |
| Minus40 | sed | mvpa | 0.16 | -0.20 | 0.51 |
| Minus40 | light | sleep | -0.70 | -1.35 | -0.06 |
| Minus40 | light | sed | -0.53 | -1.12 | 0.06 |
| Minus40 | light | mvpa | -0.37 | -0.87 | 0.12 |
| Minus40 | mvpa | sleep | -0.39 | -0.94 | 0.15 |
| Minus40 | mvpa | sed | -0.22 | -0.68 | 0.25 |
| Minus40 | mvpa | light | 0.19 | -0.26 | 0.65 |
| Minus35 | sleep | sed | 0.17 | -0.25 | 0.59 |
| Minus35 | sleep | light | 0.54 | 0.03 | 1.04 |
| Minus35 | sleep | mvpa | 0.31 | -0.11 | 0.74 |
| Minus35 | sed | sleep | -0.15 | -0.55 | 0.24 |
| Minus35 | sed | light | 0.37 | -0.06 | 0.79 |
| Minus35 | sed | mvpa | 0.14 | -0.17 | 0.45 |
| Minus35 | light | sleep | -0.61 | -1.17 | -0.05 |
| Minus35 | light | sed | -0.45 | -0.96 | 0.05 |
| Minus35 | light | mvpa | -0.32 | -0.74 | 0.11 |
| Minus35 | mvpa | sleep | -0.34 | -0.81 | 0.13 |
| Minus35 | mvpa | sed | -0.18 | -0.58 | 0.21 |
| Minus35 | mvpa | light | 0.18 | -0.22 | 0.58 |
| Minus30 | sleep | sed | 0.15 | -0.21 | 0.50 |
| Minus30 | sleep | light | 0.46 | 0.03 | 0.90 |
| Minus30 | sleep | mvpa | 0.27 | -0.10 | 0.63 |
| Minus30 | sed | sleep | -0.13 | -0.47 | 0.21 |
| Minus30 | sed | light | 0.32 | -0.05 | 0.69 |
| Minus30 | sed | mvpa | 0.12 | -0.15 | 0.39 |
| Minus30 | light | sleep | -0.52 | -0.99 | -0.04 |
| Minus30 | light | sed | -0.38 | -0.81 | 0.05 |
| Minus30 | light | mvpa | -0.26 | -0.62 | 0.10 |
| Minus30 | mvpa | sleep | -0.29 | -0.68 | 0.11 |
| Minus30 | mvpa | sed | -0.15 | -0.48 | 0.18 |
| Minus30 | mvpa | light | 0.16 | -0.18 | 0.50 |
| Minus25 | sleep | sed | 0.12 | -0.17 | 0.42 |
| Minus25 | sleep | light | 0.39 | 0.02 | 0.75 |
| Minus25 | sleep | mvpa | 0.22 | -0.08 | 0.53 |
| Minus25 | sed | sleep | -0.11 | -0.39 | 0.17 |
| Minus25 | sed | light | 0.27 | -0.04 | 0.58 |
| Minus25 | sed | mvpa | 0.10 | -0.13 | 0.33 |
| Minus25 | light | sleep | -0.42 | -0.82 | -0.03 |
| Minus25 | light | sed | -0.31 | -0.66 | 0.04 |
| Minus25 | light | mvpa | -0.21 | -0.51 | 0.09 |
| Minus25 | mvpa | sleep | -0.24 | -0.56 | 0.09 |
| Minus25 | mvpa | sed | -0.12 | -0.39 | 0.14 |
| Minus25 | mvpa | light | 0.14 | -0.14 | 0.42 |
| Minus20 | sleep | sed | 0.10 | -0.14 | 0.33 |
| Minus20 | sleep | light | 0.31 | 0.02 | 0.61 |
| Minus20 | sleep | mvpa | 0.18 | -0.07 | 0.42 |
| Minus20 | sed | sleep | -0.09 | -0.32 | 0.14 |
| Minus20 | sed | light | 0.22 | -0.03 | 0.47 |
| Minus20 | sed | mvpa | 0.08 | -0.10 | 0.27 |
| Minus20 | light | sleep | -0.34 | -0.65 | -0.03 |
| Minus20 | light | sed | -0.25 | -0.52 | 0.03 |
| Minus20 | light | mvpa | -0.16 | -0.40 | 0.07 |
| Minus20 | mvpa | sleep | -0.19 | -0.45 | 0.07 |
| Minus20 | mvpa | sed | -0.10 | -0.31 | 0.11 |
| Minus20 | mvpa | light | 0.12 | -0.11 | 0.34 |
| Minus15 | sleep | sed | 0.07 | -0.10 | 0.25 |
| Minus15 | sleep | light | 0.24 | 0.02 | 0.46 |
| Minus15 | sleep | mvpa | 0.13 | -0.05 | 0.32 |
| Minus15 | sed | sleep | -0.07 | -0.24 | 0.10 |
| Minus15 | sed | light | 0.17 | -0.02 | 0.36 |
| Minus15 | sed | mvpa | 0.06 | -0.08 | 0.20 |
| Minus15 | light | sleep | -0.25 | -0.48 | -0.02 |
| Minus15 | light | sed | -0.18 | -0.39 | 0.02 |
| Minus15 | light | mvpa | -0.12 | -0.29 | 0.06 |
| Minus15 | mvpa | sleep | -0.14 | -0.33 | 0.05 |
| Minus15 | mvpa | sed | -0.07 | -0.23 | 0.08 |
| Minus15 | mvpa | light | 0.09 | -0.07 | 0.26 |
| Minus10 | sleep | sed | 0.05 | -0.07 | 0.16 |
| Minus10 | sleep | light | 0.16 | 0.01 | 0.31 |
| Minus10 | sleep | mvpa | 0.09 | -0.03 | 0.21 |
| Minus10 | sed | sleep | -0.05 | -0.16 | 0.07 |
| Minus10 | sed | light | 0.11 | -0.02 | 0.24 |
| Minus10 | sed | mvpa | 0.04 | -0.05 | 0.14 |
| Minus10 | light | sleep | -0.16 | -0.32 | -0.01 |
| Minus10 | light | sed | -0.12 | -0.25 | 0.02 |
| Minus10 | light | mvpa | -0.08 | -0.19 | 0.04 |
| Minus10 | mvpa | sleep | -0.09 | -0.22 | 0.03 |
| Minus10 | mvpa | sed | -0.05 | -0.15 | 0.06 |
| Minus10 | mvpa | light | 0.07 | -0.05 | 0.18 |
| Minus5 | sleep | sed | 0.02 | -0.03 | 0.08 |
| Minus5 | sleep | light | 0.08 | 0.01 | 0.15 |
| Minus5 | sleep | mvpa | 0.05 | -0.02 | 0.11 |
| Minus5 | sed | sleep | -0.02 | -0.08 | 0.03 |
| Minus5 | sed | light | 0.06 | -0.01 | 0.12 |
| Minus5 | sed | mvpa | 0.02 | -0.03 | 0.07 |
| Minus5 | light | sleep | -0.08 | -0.16 | -0.01 |
| Minus5 | light | sed | -0.06 | -0.13 | 0.01 |
| Minus5 | light | mvpa | -0.04 | -0.09 | 0.02 |
| Minus5 | mvpa | sleep | -0.05 | -0.11 | 0.02 |
| Minus5 | mvpa | sed | -0.02 | -0.07 | 0.03 |
| Minus5 | mvpa | light | 0.03 | -0.02 | 0.09 |
| Plus5 | sleep | sed | -0.02 | -0.08 | 0.03 |
| Plus5 | sleep | light | -0.08 | -0.16 | -0.01 |
| Plus5 | sleep | mvpa | -0.05 | -0.11 | 0.02 |
| Plus5 | sed | sleep | 0.02 | -0.03 | 0.08 |
| Plus5 | sed | light | -0.06 | -0.13 | 0.01 |
| Plus5 | sed | mvpa | -0.02 | -0.07 | 0.03 |
| Plus5 | light | sleep | 0.08 | 0.01 | 0.15 |
| Plus5 | light | sed | 0.06 | -0.01 | 0.12 |
| Plus5 | light | mvpa | 0.03 | -0.02 | 0.09 |
| Plus5 | mvpa | sleep | 0.05 | -0.02 | 0.11 |
| Plus5 | mvpa | sed | 0.02 | -0.03 | 0.07 |
| Plus5 | mvpa | light | -0.04 | -0.09 | 0.02 |
| Plus10 | sleep | sed | -0.05 | -0.16 | 0.07 |
| Plus10 | sleep | light | -0.16 | -0.32 | -0.01 |
| Plus10 | sleep | mvpa | -0.09 | -0.22 | 0.03 |
| Plus10 | sed | sleep | 0.05 | -0.07 | 0.16 |
| Plus10 | sed | light | -0.12 | -0.25 | 0.02 |
| Plus10 | sed | mvpa | -0.05 | -0.15 | 0.06 |
| Plus10 | light | sleep | 0.16 | 0.01 | 0.31 |
| Plus10 | light | sed | 0.11 | -0.02 | 0.24 |
| Plus10 | light | mvpa | 0.07 | -0.05 | 0.18 |
| Plus10 | mvpa | sleep | 0.09 | -0.03 | 0.21 |
| Plus10 | mvpa | sed | 0.04 | -0.05 | 0.14 |
| Plus10 | mvpa | light | -0.08 | -0.19 | 0.04 |
| Plus15 | sleep | sed | -0.07 | -0.24 | 0.10 |
| Plus15 | sleep | light | -0.25 | -0.48 | -0.02 |
| Plus15 | sleep | mvpa | -0.14 | -0.33 | 0.05 |
| Plus15 | sed | sleep | 0.07 | -0.10 | 0.25 |
| Plus15 | sed | light | -0.18 | -0.39 | 0.02 |
| Plus15 | sed | mvpa | -0.07 | -0.23 | 0.08 |
| Plus15 | light | sleep | 0.24 | 0.02 | 0.46 |
| Plus15 | light | sed | 0.17 | -0.02 | 0.36 |
| Plus15 | light | mvpa | 0.09 | -0.07 | 0.26 |
| Plus15 | mvpa | sleep | 0.13 | -0.05 | 0.32 |
| Plus15 | mvpa | sed | 0.06 | -0.08 | 0.20 |
| Plus15 | mvpa | light | -0.12 | -0.29 | 0.06 |
| Plus20 | sleep | sed | -0.09 | -0.32 | 0.14 |
| Plus20 | sleep | light | -0.34 | -0.65 | -0.03 |
| Plus20 | sleep | mvpa | -0.19 | -0.45 | 0.07 |
| Plus20 | sed | sleep | 0.10 | -0.14 | 0.33 |
| Plus20 | sed | light | -0.25 | -0.52 | 0.03 |
| Plus20 | sed | mvpa | -0.10 | -0.31 | 0.11 |
| Plus20 | light | sleep | 0.31 | 0.02 | 0.61 |
| Plus20 | light | sed | 0.22 | -0.03 | 0.47 |
| Plus20 | light | mvpa | 0.12 | -0.11 | 0.34 |
| Plus20 | mvpa | sleep | 0.18 | -0.07 | 0.42 |
| Plus20 | mvpa | sed | 0.08 | -0.10 | 0.27 |
| Plus20 | mvpa | light | -0.16 | -0.40 | 0.07 |
| Plus25 | sleep | sed | -0.11 | -0.39 | 0.17 |
| Plus25 | sleep | light | -0.42 | -0.82 | -0.03 |
| Plus25 | sleep | mvpa | -0.24 | -0.56 | 0.09 |
| Plus25 | sed | sleep | 0.12 | -0.17 | 0.42 |
| Plus25 | sed | light | -0.31 | -0.66 | 0.04 |
| Plus25 | sed | mvpa | -0.12 | -0.39 | 0.14 |
| Plus25 | light | sleep | 0.39 | 0.02 | 0.75 |
| Plus25 | light | sed | 0.27 | -0.04 | 0.58 |
| Plus25 | light | mvpa | 0.14 | -0.14 | 0.42 |
| Plus25 | mvpa | sleep | 0.22 | -0.08 | 0.53 |
| Plus25 | mvpa | sed | 0.10 | -0.13 | 0.33 |
| Plus25 | mvpa | light | -0.21 | -0.51 | 0.09 |
| Plus30 | sleep | sed | -0.13 | -0.47 | 0.21 |
| Plus30 | sleep | light | -0.52 | -0.99 | -0.04 |
| Plus30 | sleep | mvpa | -0.29 | -0.68 | 0.11 |
| Plus30 | sed | sleep | 0.15 | -0.21 | 0.50 |
| Plus30 | sed | light | -0.38 | -0.81 | 0.05 |
| Plus30 | sed | mvpa | -0.15 | -0.48 | 0.18 |
| Plus30 | light | sleep | 0.46 | 0.03 | 0.90 |
| Plus30 | light | sed | 0.32 | -0.05 | 0.69 |
| Plus30 | light | mvpa | 0.16 | -0.18 | 0.50 |
| Plus30 | mvpa | sleep | 0.27 | -0.10 | 0.63 |
| Plus30 | mvpa | sed | 0.12 | -0.15 | 0.39 |
| Plus30 | mvpa | light | -0.26 | -0.62 | 0.10 |
| Plus35 | sleep | sed | -0.15 | -0.55 | 0.24 |
| Plus35 | sleep | light | -0.61 | -1.17 | -0.05 |
| Plus35 | sleep | mvpa | -0.34 | -0.81 | 0.13 |
| Plus35 | sed | sleep | 0.17 | -0.25 | 0.59 |
| Plus35 | sed | light | -0.45 | -0.96 | 0.05 |
| Plus35 | sed | mvpa | -0.18 | -0.58 | 0.21 |
| Plus35 | light | sleep | 0.54 | 0.03 | 1.04 |
| Plus35 | light | sed | 0.37 | -0.06 | 0.79 |
| Plus35 | light | mvpa | 0.18 | -0.22 | 0.58 |
| Plus35 | mvpa | sleep | 0.31 | -0.11 | 0.74 |
| Plus35 | mvpa | sed | 0.14 | -0.17 | 0.45 |
| Plus35 | mvpa | light | -0.32 | -0.74 | 0.11 |
| Plus40 | sleep | sed | -0.17 | -0.62 | 0.27 |
| Plus40 | sleep | light | -0.70 | -1.35 | -0.06 |
| Plus40 | sleep | mvpa | -0.39 | -0.94 | 0.15 |
| Plus40 | sed | sleep | 0.20 | -0.28 | 0.68 |
| Plus40 | sed | light | -0.53 | -1.12 | 0.06 |
| Plus40 | sed | mvpa | -0.22 | -0.68 | 0.25 |
| Plus40 | light | sleep | 0.61 | 0.03 | 1.19 |
| Plus40 | light | sed | 0.41 | -0.07 | 0.89 |
| Plus40 | light | mvpa | 0.19 | -0.26 | 0.65 |
| Plus40 | mvpa | sleep | 0.35 | -0.13 | 0.84 |
| Plus40 | mvpa | sed | 0.16 | -0.20 | 0.51 |
| Plus40 | mvpa | light | -0.37 | -0.87 | 0.12 |
| Plus45 | sleep | sed | -0.19 | -0.70 | 0.31 |
| Plus45 | sleep | light | -0.80 | -1.54 | -0.07 |
| Plus45 | sleep | mvpa | -0.45 | -1.07 | 0.18 |
| Plus45 | sed | sleep | 0.23 | -0.32 | 0.77 |
| Plus45 | sed | light | -0.61 | -1.28 | 0.07 |
| Plus45 | sed | mvpa | -0.25 | -0.79 | 0.28 |
| Plus45 | light | sleep | 0.68 | 0.04 | 1.33 |
| Plus45 | light | sed | 0.46 | -0.08 | 0.99 |
| Plus45 | light | mvpa | 0.21 | -0.32 | 0.73 |
| Plus45 | mvpa | sleep | 0.40 | -0.14 | 0.94 |
| Plus45 | mvpa | sed | 0.17 | -0.22 | 0.57 |
| Plus45 | mvpa | light | -0.44 | -1.00 | 0.13 |
| Plus50 | sleep | sed | -0.21 | -0.77 | 0.34 |
| Plus50 | sleep | light | -0.91 | -1.74 | -0.08 |
| Plus50 | sleep | mvpa | -0.50 | -1.21 | 0.20 |
| Plus50 | sed | sleep | 0.25 | -0.35 | 0.86 |
| Plus50 | sed | light | -0.69 | -1.45 | 0.07 |
| Plus50 | sed | mvpa | -0.29 | -0.90 | 0.32 |
| Plus50 | light | sleep | 0.76 | 0.04 | 1.47 |
| Plus50 | light | sed | 0.50 | -0.09 | 1.09 |
| Plus50 | light | mvpa | 0.21 | -0.38 | 0.80 |
| Plus50 | mvpa | sleep | 0.44 | -0.16 | 1.05 |
| Plus50 | mvpa | sed | 0.19 | -0.24 | 0.62 |
| Plus50 | mvpa | light | -0.50 | -1.14 | 0.14 |
| Plus55 | sleep | sed | -0.23 | -0.84 | 0.38 |
| Plus55 | sleep | light | -1.01 | -1.94 | -0.09 |
| Plus55 | sleep | mvpa | -0.56 | -1.36 | 0.23 |
| Plus55 | sed | sleep | 0.28 | -0.39 | 0.95 |
| Plus55 | sed | light | -0.78 | -1.63 | 0.08 |
| Plus55 | sed | mvpa | -0.33 | -1.02 | 0.37 |
| Plus55 | light | sleep | 0.83 | 0.04 | 1.61 |
| Plus55 | light | sed | 0.55 | -0.10 | 1.19 |
| Plus55 | light | mvpa | 0.22 | -0.44 | 0.88 |
| Plus55 | mvpa | sleep | 0.49 | -0.18 | 1.15 |
| Plus55 | mvpa | sed | 0.21 | -0.26 | 0.68 |
| Plus55 | mvpa | light | -0.57 | -1.29 | 0.15 |
| Plus60 | sleep | sed | -0.25 | -0.92 | 0.41 |
| Plus60 | sleep | light | -1.12 | -2.15 | -0.10 |
| Plus60 | sleep | mvpa | -0.63 | -1.51 | 0.26 |
| Plus60 | sed | sleep | 0.31 | -0.43 | 1.05 |
| Plus60 | sed | light | -0.87 | -1.82 | 0.09 |
| Plus60 | sed | mvpa | -0.37 | -1.15 | 0.41 |
| Plus60 | light | sleep | 0.90 | 0.04 | 1.75 |
| Plus60 | light | sed | 0.59 | -0.11 | 1.29 |
| Plus60 | light | mvpa | 0.22 | -0.52 | 0.95 |
| Plus60 | mvpa | sleep | 0.53 | -0.19 | 1.25 |
| Plus60 | mvpa | sed | 0.22 | -0.29 | 0.73 |
| Plus60 | mvpa | light | -0.65 | -1.45 | 0.15 |

**Model 3**

| **Time** | **comp+** | **comp-** | **delta_pred** | **ci_lo** | **ci_up** |
| --- | --- | --- | --- | --- | --- |
| Minus60 | sleep | sed | 0.31 | -0.46 | 1.09 |
| Minus60 | sleep | light | 0.78 | -0.13 | 1.69 |
| Minus60 | sleep | mvpa | 0.46 | -0.31 | 1.24 |
| Minus60 | sed | sleep | -0.26 | -0.96 | 0.44 |
| Minus60 | sed | light | 0.47 | -0.28 | 1.21 |
| Minus60 | sed | mvpa | 0.15 | -0.40 | 0.69 |
| Minus60 | light | sleep | -0.96 | -2.05 | 0.13 |
| Minus60 | light | sed | -0.70 | -1.72 | 0.32 |
| Minus60 | light | mvpa | -0.55 | -1.40 | 0.30 |
| Minus60 | mvpa | sleep | -0.52 | -1.48 | 0.44 |
| Minus60 | mvpa | sed | -0.26 | -1.11 | 0.58 |
| Minus60 | mvpa | light | 0.21 | -0.58 | 0.99 |
| Minus55 | sleep | sed | 0.29 | -0.42 | 1.00 |
| Minus55 | sleep | light | 0.72 | -0.12 | 1.56 |
| Minus55 | sleep | mvpa | 0.42 | -0.29 | 1.13 |
| Minus55 | sed | sleep | -0.24 | -0.89 | 0.40 |
| Minus55 | sed | light | 0.43 | -0.26 | 1.12 |
| Minus55 | sed | mvpa | 0.14 | -0.37 | 0.64 |
| Minus55 | light | sleep | -0.87 | -1.85 | 0.12 |
| Minus55 | light | sed | -0.62 | -1.54 | 0.29 |
| Minus55 | light | mvpa | -0.49 | -1.25 | 0.27 |
| Minus55 | mvpa | sleep | -0.47 | -1.33 | 0.39 |
| Minus55 | mvpa | sed | -0.23 | -0.98 | 0.52 |
| Minus55 | mvpa | light | 0.20 | -0.50 | 0.91 |
| Minus50 | sleep | sed | 0.26 | -0.38 | 0.90 |
| Minus50 | sleep | light | 0.66 | -0.10 | 1.42 |
| Minus50 | sleep | mvpa | 0.38 | -0.26 | 1.03 |
| Minus50 | sed | sleep | -0.22 | -0.81 | 0.37 |
| Minus50 | sed | light | 0.40 | -0.23 | 1.03 |
| Minus50 | sed | mvpa | 0.13 | -0.34 | 0.59 |
| Minus50 | light | sleep | -0.78 | -1.66 | 0.11 |
| Minus50 | light | sed | -0.55 | -1.37 | 0.26 |
| Minus50 | light | mvpa | -0.43 | -1.11 | 0.25 |
| Minus50 | mvpa | sleep | -0.42 | -1.19 | 0.34 |
| Minus50 | mvpa | sed | -0.20 | -0.86 | 0.46 |
| Minus50 | mvpa | light | 0.20 | -0.43 | 0.83 |
| Minus45 | sleep | sed | 0.23 | -0.34 | 0.80 |
| Minus45 | sleep | light | 0.59 | -0.09 | 1.28 |
| Minus45 | sleep | mvpa | 0.35 | -0.24 | 0.93 |
| Minus45 | sed | sleep | -0.20 | -0.73 | 0.33 |
| Minus45 | sed | light | 0.36 | -0.21 | 0.94 |
| Minus45 | sed | mvpa | 0.12 | -0.31 | 0.54 |
| Minus45 | light | sleep | -0.69 | -1.47 | 0.10 |
| Minus45 | light | sed | -0.49 | -1.21 | 0.23 |
| Minus45 | light | mvpa | -0.37 | -0.97 | 0.23 |
| Minus45 | mvpa | sleep | -0.38 | -1.05 | 0.30 |
| Minus45 | mvpa | sed | -0.18 | -0.75 | 0.40 |
| Minus45 | mvpa | light | 0.19 | -0.37 | 0.75 |
| Minus40 | sleep | sed | 0.20 | -0.31 | 0.71 |
| Minus40 | sleep | light | 0.53 | -0.08 | 1.14 |
| Minus40 | sleep | mvpa | 0.31 | -0.21 | 0.83 |
| Minus40 | sed | sleep | -0.18 | -0.65 | 0.29 |
| Minus40 | sed | light | 0.33 | -0.19 | 0.84 |
| Minus40 | sed | mvpa | 0.11 | -0.27 | 0.48 |
| Minus40 | light | sleep | -0.60 | -1.29 | 0.08 |
| Minus40 | light | sed | -0.43 | -1.06 | 0.21 |
| Minus40 | light | mvpa | -0.32 | -0.84 | 0.20 |
| Minus40 | mvpa | sleep | -0.33 | -0.92 | 0.26 |
| Minus40 | mvpa | sed | -0.15 | -0.65 | 0.35 |
| Minus40 | mvpa | light | 0.18 | -0.31 | 0.67 |
| Minus35 | sleep | sed | 0.18 | -0.27 | 0.62 |
| Minus35 | sleep | light | 0.47 | -0.07 | 1.00 |
| Minus35 | sleep | mvpa | 0.27 | -0.18 | 0.72 |
| Minus35 | sed | sleep | -0.16 | -0.57 | 0.26 |
| Minus35 | sed | light | 0.29 | -0.16 | 0.75 |
| Minus35 | sed | mvpa | 0.09 | -0.24 | 0.43 |
| Minus35 | light | sleep | -0.52 | -1.12 | 0.07 |
| Minus35 | light | sed | -0.36 | -0.91 | 0.18 |
| Minus35 | light | mvpa | -0.27 | -0.72 | 0.18 |
| Minus35 | mvpa | sleep | -0.29 | -0.79 | 0.22 |
| Minus35 | mvpa | sed | -0.13 | -0.55 | 0.30 |
| Minus35 | mvpa | light | 0.16 | -0.26 | 0.59 |
| Minus30 | sleep | sed | 0.15 | -0.23 | 0.53 |
| Minus30 | sleep | light | 0.40 | -0.06 | 0.87 |
| Minus30 | sleep | mvpa | 0.23 | -0.16 | 0.62 |
| Minus30 | sed | sleep | -0.14 | -0.49 | 0.22 |
| Minus30 | sed | light | 0.25 | -0.14 | 0.65 |
| Minus30 | sed | mvpa | 0.08 | -0.21 | 0.37 |
| Minus30 | light | sleep | -0.44 | -0.95 | 0.06 |
| Minus30 | light | sed | -0.31 | -0.77 | 0.15 |
| Minus30 | light | mvpa | -0.22 | -0.61 | 0.16 |
| Minus30 | mvpa | sleep | -0.24 | -0.67 | 0.18 |
| Minus30 | mvpa | sed | -0.11 | -0.46 | 0.25 |
| Minus30 | mvpa | light | 0.15 | -0.21 | 0.51 |
| Minus25 | sleep | sed | 0.12 | -0.19 | 0.44 |
| Minus25 | sleep | light | 0.34 | -0.05 | 0.72 |
| Minus25 | sleep | mvpa | 0.19 | -0.13 | 0.52 |
| Minus25 | sed | sleep | -0.11 | -0.41 | 0.18 |
| Minus25 | sed | light | 0.21 | -0.12 | 0.55 |
| Minus25 | sed | mvpa | 0.07 | -0.18 | 0.32 |
| Minus25 | light | sleep | -0.37 | -0.78 | 0.05 |
| Minus25 | light | sed | -0.25 | -0.63 | 0.13 |
| Minus25 | light | mvpa | -0.18 | -0.50 | 0.13 |
| Minus25 | mvpa | sleep | -0.20 | -0.55 | 0.15 |
| Minus25 | mvpa | sed | -0.09 | -0.38 | 0.20 |
| Minus25 | mvpa | light | 0.13 | -0.17 | 0.43 |
| Minus20 | sleep | sed | 0.10 | -0.15 | 0.35 |
| Minus20 | sleep | light | 0.27 | -0.04 | 0.58 |
| Minus20 | sleep | mvpa | 0.15 | -0.11 | 0.42 |
| Minus20 | sed | sleep | -0.09 | -0.33 | 0.15 |
| Minus20 | sed | light | 0.17 | -0.10 | 0.44 |
| Minus20 | sed | mvpa | 0.06 | -0.14 | 0.26 |
| Minus20 | light | sleep | -0.29 | -0.62 | 0.04 |
| Minus20 | light | sed | -0.20 | -0.49 | 0.10 |
| Minus20 | light | mvpa | -0.14 | -0.39 | 0.11 |
| Minus20 | mvpa | sleep | -0.16 | -0.44 | 0.12 |
| Minus20 | mvpa | sed | -0.07 | -0.29 | 0.16 |
| Minus20 | mvpa | light | 0.11 | -0.13 | 0.34 |
| Minus15 | sleep | sed | 0.07 | -0.11 | 0.26 |
| Minus15 | sleep | light | 0.20 | -0.03 | 0.44 |
| Minus15 | sleep | mvpa | 0.12 | -0.08 | 0.31 |
| Minus15 | sed | sleep | -0.07 | -0.25 | 0.11 |
| Minus15 | sed | light | 0.13 | -0.07 | 0.34 |
| Minus15 | sed | mvpa | 0.04 | -0.11 | 0.19 |
| Minus15 | light | sleep | -0.21 | -0.46 | 0.03 |
| Minus15 | light | sed | -0.15 | -0.36 | 0.07 |
| Minus15 | light | mvpa | -0.10 | -0.29 | 0.08 |
| Minus15 | mvpa | sleep | -0.12 | -0.33 | 0.09 |
| Minus15 | mvpa | sed | -0.05 | -0.22 | 0.12 |
| Minus15 | mvpa | light | 0.08 | -0.10 | 0.26 |
| Minus10 | sleep | sed | 0.05 | -0.08 | 0.17 |
| Minus10 | sleep | light | 0.14 | -0.02 | 0.30 |
| Minus10 | sleep | mvpa | 0.08 | -0.05 | 0.21 |
| Minus10 | sed | sleep | -0.05 | -0.17 | 0.07 |
| Minus10 | sed | light | 0.09 | -0.05 | 0.23 |
| Minus10 | sed | mvpa | 0.03 | -0.07 | 0.13 |
| Minus10 | light | sleep | -0.14 | -0.30 | 0.02 |
| Minus10 | light | sed | -0.10 | -0.24 | 0.05 |
| Minus10 | light | mvpa | -0.07 | -0.19 | 0.06 |
| Minus10 | mvpa | sleep | -0.08 | -0.22 | 0.06 |
| Minus10 | mvpa | sed | -0.03 | -0.14 | 0.08 |
| Minus10 | mvpa | light | 0.06 | -0.06 | 0.18 |
| Minus5 | sleep | sed | 0.02 | -0.04 | 0.09 |
| Minus5 | sleep | light | 0.07 | -0.01 | 0.15 |
| Minus5 | sleep | mvpa | 0.04 | -0.03 | 0.11 |
| Minus5 | sed | sleep | -0.02 | -0.08 | 0.04 |
| Minus5 | sed | light | 0.05 | -0.02 | 0.11 |
| Minus5 | sed | mvpa | 0.01 | -0.04 | 0.07 |
| Minus5 | light | sleep | -0.07 | -0.15 | 0.01 |
| Minus5 | light | sed | -0.05 | -0.12 | 0.02 |
| Minus5 | light | mvpa | -0.03 | -0.09 | 0.03 |
| Minus5 | mvpa | sleep | -0.04 | -0.11 | 0.03 |
| Minus5 | mvpa | sed | -0.02 | -0.07 | 0.04 |
| Minus5 | mvpa | light | 0.03 | -0.03 | 0.09 |
| Plus5 | sleep | sed | -0.02 | -0.08 | 0.04 |
| Plus5 | sleep | light | -0.07 | -0.15 | 0.01 |
| Plus5 | sleep | mvpa | -0.04 | -0.11 | 0.03 |
| Plus5 | sed | sleep | 0.02 | -0.04 | 0.09 |
| Plus5 | sed | light | -0.05 | -0.12 | 0.02 |
| Plus5 | sed | mvpa | -0.02 | -0.07 | 0.04 |
| Plus5 | light | sleep | 0.07 | -0.01 | 0.15 |
| Plus5 | light | sed | 0.05 | -0.02 | 0.11 |
| Plus5 | light | mvpa | 0.03 | -0.03 | 0.09 |
| Plus5 | mvpa | sleep | 0.04 | -0.03 | 0.11 |
| Plus5 | mvpa | sed | 0.01 | -0.04 | 0.07 |
| Plus5 | mvpa | light | -0.03 | -0.09 | 0.03 |
| Plus10 | sleep | sed | -0.05 | -0.17 | 0.07 |
| Plus10 | sleep | light | -0.14 | -0.30 | 0.02 |
| Plus10 | sleep | mvpa | -0.08 | -0.22 | 0.06 |
| Plus10 | sed | sleep | 0.05 | -0.08 | 0.17 |
| Plus10 | sed | light | -0.10 | -0.24 | 0.05 |
| Plus10 | sed | mvpa | -0.03 | -0.14 | 0.08 |
| Plus10 | light | sleep | 0.14 | -0.02 | 0.30 |
| Plus10 | light | sed | 0.09 | -0.05 | 0.23 |
| Plus10 | light | mvpa | 0.06 | -0.06 | 0.18 |
| Plus10 | mvpa | sleep | 0.08 | -0.05 | 0.21 |
| Plus10 | mvpa | sed | 0.03 | -0.07 | 0.13 |
| Plus10 | mvpa | light | -0.07 | -0.19 | 0.06 |
| Plus15 | sleep | sed | -0.07 | -0.25 | 0.11 |
| Plus15 | sleep | light | -0.21 | -0.46 | 0.03 |
| Plus15 | sleep | mvpa | -0.12 | -0.33 | 0.09 |
| Plus15 | sed | sleep | 0.07 | -0.11 | 0.26 |
| Plus15 | sed | light | -0.15 | -0.36 | 0.07 |
| Plus15 | sed | mvpa | -0.05 | -0.22 | 0.12 |
| Plus15 | light | sleep | 0.20 | -0.03 | 0.44 |
| Plus15 | light | sed | 0.13 | -0.07 | 0.34 |
| Plus15 | light | mvpa | 0.08 | -0.10 | 0.26 |
| Plus15 | mvpa | sleep | 0.12 | -0.08 | 0.31 |
| Plus15 | mvpa | sed | 0.04 | -0.11 | 0.19 |
| Plus15 | mvpa | light | -0.10 | -0.29 | 0.08 |
| Plus20 | sleep | sed | -0.09 | -0.33 | 0.15 |
| Plus20 | sleep | light | -0.29 | -0.62 | 0.04 |
| Plus20 | sleep | mvpa | -0.16 | -0.44 | 0.12 |
| Plus20 | sed | sleep | 0.10 | -0.15 | 0.35 |
| Plus20 | sed | light | -0.20 | -0.49 | 0.10 |
| Plus20 | sed | mvpa | -0.07 | -0.29 | 0.16 |
| Plus20 | light | sleep | 0.27 | -0.04 | 0.58 |
| Plus20 | light | sed | 0.17 | -0.10 | 0.44 |
| Plus20 | light | mvpa | 0.11 | -0.13 | 0.34 |
| Plus20 | mvpa | sleep | 0.15 | -0.11 | 0.42 |
| Plus20 | mvpa | sed | 0.06 | -0.14 | 0.26 |
| Plus20 | mvpa | light | -0.14 | -0.39 | 0.11 |
| Plus25 | sleep | sed | -0.11 | -0.41 | 0.18 |
| Plus25 | sleep | light | -0.37 | -0.78 | 0.05 |
| Plus25 | sleep | mvpa | -0.20 | -0.55 | 0.15 |
| Plus25 | sed | sleep | 0.12 | -0.19 | 0.44 |
| Plus25 | sed | light | -0.25 | -0.63 | 0.13 |
| Plus25 | sed | mvpa | -0.09 | -0.38 | 0.20 |
| Plus25 | light | sleep | 0.34 | -0.05 | 0.72 |
| Plus25 | light | sed | 0.21 | -0.12 | 0.55 |
| Plus25 | light | mvpa | 0.13 | -0.17 | 0.43 |
| Plus25 | mvpa | sleep | 0.19 | -0.13 | 0.52 |
| Plus25 | mvpa | sed | 0.07 | -0.18 | 0.32 |
| Plus25 | mvpa | light | -0.18 | -0.50 | 0.13 |
| Plus30 | sleep | sed | -0.14 | -0.49 | 0.22 |
| Plus30 | sleep | light | -0.44 | -0.95 | 0.06 |
| Plus30 | sleep | mvpa | -0.24 | -0.67 | 0.18 |
| Plus30 | sed | sleep | 0.15 | -0.23 | 0.53 |
| Plus30 | sed | light | -0.31 | -0.77 | 0.15 |
| Plus30 | sed | mvpa | -0.11 | -0.46 | 0.25 |
| Plus30 | light | sleep | 0.40 | -0.06 | 0.87 |
| Plus30 | light | sed | 0.25 | -0.14 | 0.65 |
| Plus30 | light | mvpa | 0.15 | -0.21 | 0.51 |
| Plus30 | mvpa | sleep | 0.23 | -0.16 | 0.62 |
| Plus30 | mvpa | sed | 0.08 | -0.21 | 0.37 |
| Plus30 | mvpa | light | -0.22 | -0.61 | 0.16 |
| Plus35 | sleep | sed | -0.16 | -0.57 | 0.26 |
| Plus35 | sleep | light | -0.52 | -1.12 | 0.07 |
| Plus35 | sleep | mvpa | -0.29 | -0.79 | 0.22 |
| Plus35 | sed | sleep | 0.18 | -0.27 | 0.62 |
| Plus35 | sed | light | -0.36 | -0.91 | 0.18 |
| Plus35 | sed | mvpa | -0.13 | -0.55 | 0.30 |
| Plus35 | light | sleep | 0.47 | -0.07 | 1.00 |
| Plus35 | light | sed | 0.29 | -0.16 | 0.75 |
| Plus35 | light | mvpa | 0.16 | -0.26 | 0.59 |
| Plus35 | mvpa | sleep | 0.27 | -0.18 | 0.72 |
| Plus35 | mvpa | sed | 0.09 | -0.24 | 0.43 |
| Plus35 | mvpa | light | -0.27 | -0.72 | 0.18 |
| Plus40 | sleep | sed | -0.18 | -0.65 | 0.29 |
| Plus40 | sleep | light | -0.60 | -1.29 | 0.08 |
| Plus40 | sleep | mvpa | -0.33 | -0.92 | 0.26 |
| Plus40 | sed | sleep | 0.20 | -0.31 | 0.71 |
| Plus40 | sed | light | -0.43 | -1.06 | 0.21 |
| Plus40 | sed | mvpa | -0.15 | -0.65 | 0.35 |
| Plus40 | light | sleep | 0.53 | -0.08 | 1.14 |
| Plus40 | light | sed | 0.33 | -0.19 | 0.84 |
| Plus40 | light | mvpa | 0.18 | -0.31 | 0.67 |
| Plus40 | mvpa | sleep | 0.31 | -0.21 | 0.83 |
| Plus40 | mvpa | sed | 0.11 | -0.27 | 0.48 |
| Plus40 | mvpa | light | -0.32 | -0.84 | 0.20 |
| Plus45 | sleep | sed | -0.20 | -0.73 | 0.33 |
| Plus45 | sleep | light | -0.69 | -1.47 | 0.10 |
| Plus45 | sleep | mvpa | -0.38 | -1.05 | 0.30 |
| Plus45 | sed | sleep | 0.23 | -0.34 | 0.80 |
| Plus45 | sed | light | -0.49 | -1.21 | 0.23 |
| Plus45 | sed | mvpa | -0.18 | -0.75 | 0.40 |
| Plus45 | light | sleep | 0.59 | -0.09 | 1.28 |
| Plus45 | light | sed | 0.36 | -0.21 | 0.94 |
| Plus45 | light | mvpa | 0.19 | -0.37 | 0.75 |
| Plus45 | mvpa | sleep | 0.35 | -0.24 | 0.93 |
| Plus45 | mvpa | sed | 0.12 | -0.31 | 0.54 |
| Plus45 | mvpa | light | -0.37 | -0.97 | 0.23 |
| Plus50 | sleep | sed | -0.22 | -0.81 | 0.37 |
| Plus50 | sleep | light | -0.78 | -1.66 | 0.11 |
| Plus50 | sleep | mvpa | -0.42 | -1.19 | 0.34 |
| Plus50 | sed | sleep | 0.26 | -0.38 | 0.90 |
| Plus50 | sed | light | -0.55 | -1.37 | 0.26 |
| Plus50 | sed | mvpa | -0.20 | -0.86 | 0.46 |
| Plus50 | light | sleep | 0.66 | -0.10 | 1.42 |
| Plus50 | light | sed | 0.40 | -0.23 | 1.03 |
| Plus50 | light | mvpa | 0.20 | -0.43 | 0.83 |
| Plus50 | mvpa | sleep | 0.38 | -0.26 | 1.03 |
| Plus50 | mvpa | sed | 0.13 | -0.34 | 0.59 |
| Plus50 | mvpa | light | -0.43 | -1.11 | 0.25 |
| Plus55 | sleep | sed | -0.24 | -0.89 | 0.40 |
| Plus55 | sleep | light | -0.87 | -1.85 | 0.12 |
| Plus55 | sleep | mvpa | -0.47 | -1.33 | 0.39 |
| Plus55 | sed | sleep | 0.29 | -0.42 | 1.00 |
| Plus55 | sed | light | -0.62 | -1.54 | 0.29 |
| Plus55 | sed | mvpa | -0.23 | -0.98 | 0.52 |
| Plus55 | light | sleep | 0.72 | -0.12 | 1.56 |
| Plus55 | light | sed | 0.43 | -0.26 | 1.12 |
| Plus55 | light | mvpa | 0.20 | -0.50 | 0.91 |
| Plus55 | mvpa | sleep | 0.42 | -0.29 | 1.13 |
| Plus55 | mvpa | sed | 0.14 | -0.37 | 0.64 |
| Plus55 | mvpa | light | -0.49 | -1.25 | 0.27 |
| Plus60 | sleep | sed | -0.26 | -0.96 | 0.44 |
| Plus60 | sleep | light | -0.96 | -2.05 | 0.13 |
| Plus60 | sleep | mvpa | -0.52 | -1.48 | 0.44 |
| Plus60 | sed | sleep | 0.31 | -0.46 | 1.09 |
| Plus60 | sed | light | -0.70 | -1.72 | 0.32 |
| Plus60 | sed | mvpa | -0.26 | -1.11 | 0.58 |
| Plus60 | light | sleep | 0.78 | -0.13 | 1.69 |
| Plus60 | light | sed | 0.47 | -0.28 | 1.21 |
| Plus60 | light | mvpa | 0.21 | -0.58 | 0.99 |
| Plus60 | mvpa | sleep | 0.46 | -0.31 | 1.24 |
| Plus60 | mvpa | sed | 0.15 | -0.40 | 0.69 |
| Plus60 | mvpa | light | -0.55 | -1.40 | 0.30 |
